# Dual-color dual-focus line-scanning FCS for quantitative analysis of receptor-ligand interactions in living specimens

**DOI:** 10.1038/srep10149

**Published:** 2015-05-07

**Authors:** René M. Dörlich, Qing Chen, Per Niklas Hedde, Vittoria Schuster, Marc Hippler, Janine Wesslowski, Gary Davidson, G. Ulrich Nienhaus

**Affiliations:** 1 Institute of Applied Physics, Karlsruhe Institute of Technology (KIT), 76049 Karlsruhe, Germany; 2 Institute of Toxicology and Genetics, Karlsruhe Institute of Technology (KIT), 76021 Karlsruhe, Germany; 3 Faculty of Bioscience, University of Heidelberg, 69120 Heidelberg, Germany; 4 Department of Physics, University of Illinois at Urbana-Champaign, Urbana, IL 61801, USA

## Abstract

Cellular communication in multi-cellular organisms is mediated to a large extent by a multitude of cell-surface receptors that bind specific ligands. An in-depth understanding of cell signaling networks requires quantitative information on ligand-receptor interactions within living systems. In principle, fluorescence correlation spectroscopy (FCS) based methods can provide such data, but live-cell applications have proven extremely challenging. Here, we have developed an integrated dual-color dual-focus line-scanning fluorescence correlation spectroscopy (2c2f lsFCS) technique that greatly facilitates live-cell and tissue experiments. Absolute ligand and receptor concentrations and their diffusion coefficients within the cell membrane can be quantified without the need to perform additional calibration experiments. We also determine the concentration of ligands diffusing in the medium outside the cell within the same experiment by using a raster image correlation spectroscopy (RICS) based analysis. We have applied this robust technique to study the interactions of two Wnt antagonists, Dickkopf1 and Dickkopf2 (Dkk1/2), to their cognate receptor, low-density-lipoprotein-receptor related protein 6 (LRP6), in the plasma membrane of living HEK293T cells. We obtained significantly lower affinities than previously reported using in vitro studies, underscoring the need to measure such data on living cells or tissues.

Cellular communication is crucial for the development and homeostasis of multicellular organisms. A variety of specific cell signaling pathways exist which involve binding of secreted extracellular ligands to their cognate receptors, usually located on the surfaces of responding cells. An in-depth study of the activation of specific cell signaling pathways at the molecular level requires a quantitative assessment of the receptor-ligand complexes formed on the signal-receiving cell, which depends on local concentrations and the binding affinity of the ligand-receptor pairs. The affinity, quantified by the equilibrium dissociation coefficient, *K*_*d*_, is a key parameter for the specificity of signaling pathways and provides important information for pharmaceutical drug screening. Many biochemical and biophysical *in-vitro* assays have been developed for the quantification of ligand-receptor interactions^1^. Frequently, however, the proteins cannot be prepared in sufficient quantity and purity for these techniques. Moreover, the results from *in-vitro* studies can differ markedly from those obtained under *in-vivo* conditions due to the complex environment of living cells and tissues^2^.

It is clearly important to study ligand-receptor interactions directly in living cells, tissues and indeed entire organisms. To this end, fluorescence correlation spectroscopy (FCS) has emerged as a powerful biophysical technique^3^. Analyzing intensity fluctuations of light emitted by fluorescent molecules diffusing through a minute observation volume (≤10^−15^ l) allows concentration and diffusion coefficients to be precisely determined. Usually, a confocal laser scanning microscope is used to position the observation volume in the sample and to detect fluorescently labeled molecules diffusing through it. From the recorded intensity time traces, the autocorrelation function, *G*(*T*), is computed for different lag times, *T* Its amplitude at time zero, *G*(0), yields the fluorophore concentration; its temporal decay, *i.e.*, the correlation time, *T*_*D*_, is inversely related to the diffusion coefficient, *D* (Supplementary Information, Text S1 and Fig. S1). The size of the observation volume is usually obtained via a reference measurement using a fluorophore with a known diffusion coefficient at nanomolar concentration.

Techniques employed in conventional FCS are increasingly applied to live cell and tissue/organism experiments^3^, *e.g.*, to determine equilibrium dissociation coefficients of biomolecular interactions in live yeast^4^ and zebrafish^5^ samples. For the work presented here, three of these techniques are relevant and used in combination. (i) Dual-focus FCS, where intensity fluctuations are measured from two partially overlapping regions that are separated by a known distance^6^. This displacement allows the observation volume to be determined within the experiment itself, making a separate calibration measurement obsolete. This saves time and helps avoid systematic errors in observation volume calibration when using biological samples with refractive index variations. (ii) Dual-color fluorescence cross-correlation spectroscopy (FCCS), which involves the simultaneous measurement of two differently labeled molecules in separate spectral channels^7^. A cross-correlation signal is observed if the two molecules bind to each other so that they diffuse as an entity. Although these two methods have been successfully employed on living cells^8^ and organisms^9^, they are extremely challenging because of the intrinsic movements within biological samples. For example, membrane fluctuations generate large fluorescence intensity variations that can obscure the detection of the fluctuations arising from molecules diffusing in the membrane. (iii) Line-scanning FCS, a technique introduced by Schwille and coworkers^10^ to reduce the effect from such intensity fluctuations arising from membrane movements. The FCS observation volume is repeatedly raster-scanned perpendicularly through a membrane while the intensity is recorded pixel by pixel with a fixed pixel dwell time (Supplementary Information, Text S2 and Fig. S2). The fluorescent molecules associated with the membrane give rise to intensity peaks whenever the focus intersects the membrane. The location of the peak is determined for each scan line and fluctuations from membrane movements are eliminated by alignment of the data from multiple scans. As in dual-focus FCS, a reference measurement can be avoided by scanning the focus along two parallel lines with known separation and cross-correlating the intensities from the two intersections with the membrane (Supplementary Information, Text S3 and Fig. S3). Evidently, dual-color FCCS experiments can also be combined with line scanning (Supplementary Information, Text S4 and Fig. S4). As an added benefit, alternating excitation with two colors eliminates spectral cross-talk effects.

In 2009, Ries *et al.*^2^ reported the combined use of dual-focus line scanning FCS and dual-color line scanning FCCS measurements together with static FCS in a modular way. The two FCS experiments were carried out in succession, and the subsequent analysis was performed globally to yield a single set of parameters. This work was a major advance and clearly demonstrated that receptor-ligand interactions can be quantified *in vivo* by combining these methods. Nevertheless, because dual-color and dual-focus lsFCS measurements are carried out consecutively rather than simultaneously, slow sample drift over several minutes remains a problematic issue and sample properties have to stay constant over longer periods of time to enable a global analysis of the entire set of data. Here, we present an advanced strategy that overcomes these problems. This novel approach involves the integration of all three FCS modes mentioned above in a single procedure, which we call dual-color dual-focus line-scanning FCS (2c2f lsFCS) (Fig. 1, Supplementary Information, Text S5). We demonstrate the robustness of the technique by measuring interactions between receptors and ligands involved in Wnt signaling.

## Results

**Figure 1:**
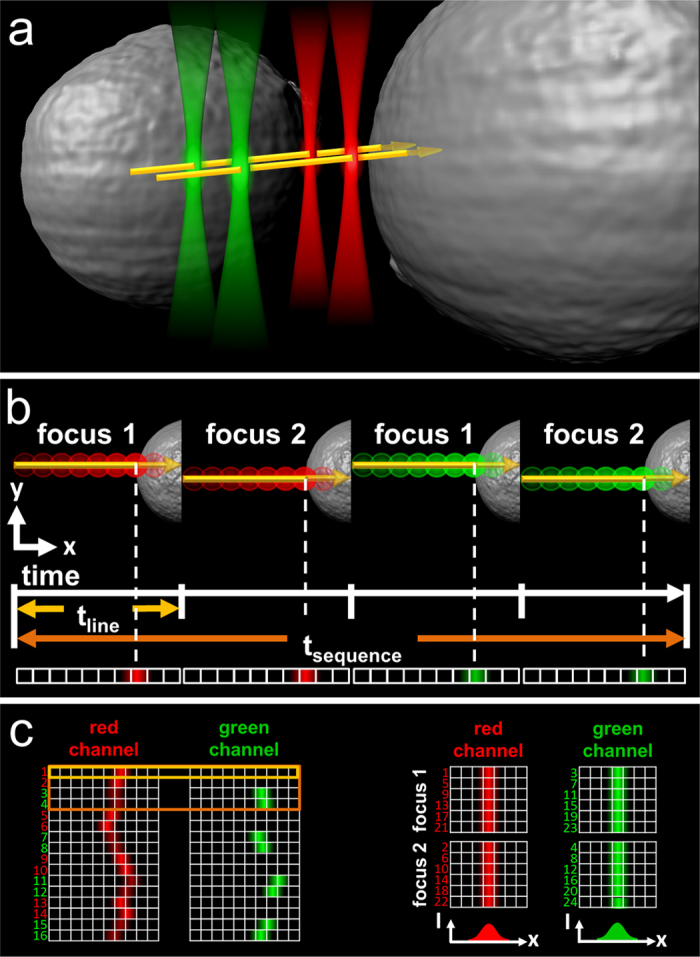
Data acquisition scheme of 2c2f lsFCS. (**a**) In a confocal laser scanning microscope, the observation focus is scanned perpendicularly through the cell membrane along two lines separated by a small, fixed distance, and the excitation light is alternated between two colors (green and red). (**b**) In a single scan of duration *t*_*line*_ (2 ms in our case), the fluorescence emission is registered separately for the two colors and binned in pixels according to their spatial position along the scan axis. A scan sequence of duration *t*_*sequence*_ consists of four sequential scans, focus 1 and 2 with red excitation, and focus 1 and 2 with green excitation, and is repeated many times. (**c**) The intensities measured in all line scans are arranged as kymograms, where the horizontal axis shows the intensity as a function of scanner position, and the vertical axis labels the scan number. Membrane fluctuations during the measurement are removed by shifting the line data horizontally to a common origin. The data are separated into four arrays corresponding to one of the four line scans of the sequence. Fluorescence intensity time traces are computed from these data, from which correlation functions are calculated and analyzed to reveal the dynamics.

**Figure 2:**
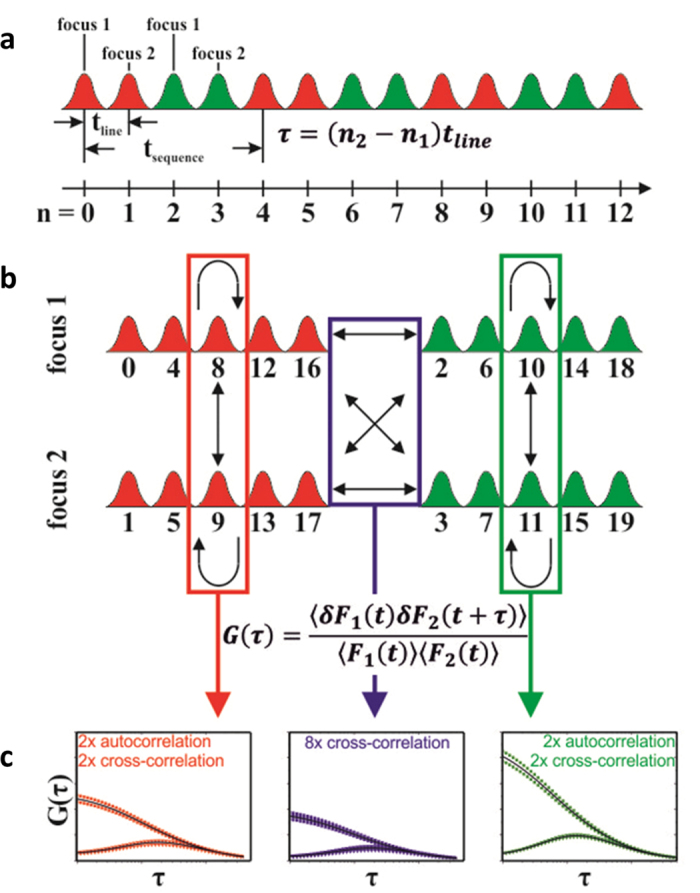
Data analysis in 2c2f lsFCS. (**a**) Line scan intensity data acquired with repetitive application of the four line scan sequence: scans 1 and 2 at focal positions 1 and 2 (red Gaussians), respectively, with red excitation and detection in the red color channel, and scans 3 and 4 at focal positions 1 and 2 (green Gaussians), respectively, with green excitation and detection in the green color channel. Each line scan is numbered by ascending *n*, so the temporal separation between any pair of lines is given by *τ* = (*n*_2_ – *n*_1_) *t*_*line*_. (**b**) The data are rearranged into four intensity time traces, and four auto- (bent arrows) and twelve cross-correlation functions (straight and double arrows) are computed. (**c**) These 16 correlation curves are globally fitted to obtain diffusion coefficients and concentrations of the two diffusing species.

For 2c2f lsFCS analysis, the following sequence of four line scans is repetitively performed many times to gather statistics: red laser in focus 1, red laser in focus 2, green laser in focus 1, and green laser in focus 2 (Fig. 1a,b). The scan data are subsequently aligned to a common time origin to remove the effect of membrane dynamics (Fig. 1c). With this four-line sequence that alternates between the sampling of two-color and two-focus correlations on a millisecond time scale, slower variations in the sample will have negligible effects. From the time sequence of the intensities (Fig. 2a), all 16 pair correlation functions are calculated (Fig. 2b,c), from which the diffusion coefficients and molecule concentrations are extracted in a global fit (Supplementary Information, Text S5). Thus, 2c2f lsFCS combines two dual-focus line-scanning FCS measurements, one for the red and one for the green channel, and two dual-color line-scanning FCS measurements (red focus 1 with green focus 1 and red focus 2 with green focus 2). Moreover, our integrated approach also enables us to analyze cross-correlations between the two parameters color and focus, *i.e.*, dual-color dual-focus cross-correlations (red focus 1 with green focus 2 and red focus 2 with green focus 1). These correlations, which are not accessible within the modular approach, further enhance the statistics and, most importantly, directly determine the effective observation volume of the dual-color experiment, which depends on the observation volumes of the green and red color channels and is required for determining the receptor-ligand complex concentration (Supplementary Information, Text S5). The interdependence of multiple correlation functions on a small set of instrument parameters yields robust results in the global fit.

In addition, we have implemented a raster image correlation (RICS^11^)-based analysis that can be applied to the 2c2f lsFCS data to determine the free ligand concentration in the extracellular space by correlating the intensities of different pixels with each other (Supplementary Information, Text S6 and Fig. S5). Thereby, an additional, conventional FCS measurement of the diffusion of a freely diffusing ligand in the extracellular space may become obsolete, resulting in a further decrease in measurement time.

We have applied our 2c2f lsFCS method to ligand-receptor interactions in the Wnt signaling pathway, which is arguably one of the most important cellular communication systems known to date. Cell-cell communication by the Wnt pathway uses multiple ligand-receptor interactions to control essential functions during development, adult homeostasis and in disease^12^. Although several ligand-receptor interactions in the Wnt pathway have been characterized by *in-vitro* methods, a robust, standardized method to quantify and compare these binding events in live cells and tissues will be of great benefit. Our 2c2f lsFCS method should be well suited for this purpose and we present here initial proof of principal experiments using a ligand-receptor pair that has been well characterized, both in terms of functional relevance for Wnt signaling as well as *in-vitro* binding affinity. This receptor-ligand pair is the Wnt co-receptor low-density-lipoprotein receptor-related protein 6 (LRP6) and its ligand, Dickkopf1 (Dkk1).

Dickkopf (Dkk) glycoproteins belong to an evolutionarily conserved four member family (Dkk1 – 4) and play important roles in metazoan development and homeostasis by inhibiting Wnt/β-catenin signaling^13^. Dkk proteins bind to the Wnt co-receptors LRP5/6 to inhibit Wnt/β-catenin signaling and can additionally associate with Kremen receptors to further modulate signaling^13^. Biologically, Dkks play essential roles during embryonic head and limb formation and regulation of bone density in the adult. They are furthermore implicated in cancer and Alzheimer’s disease^13^. Dkk1 has been reported to bind to LRP6 with an equilibrium dissociation coefficient, *K*_d_, in the range of 0.3 to 0.5 nM, as determined by *in-vitro* studies^14^,^15^,^16^,^17^. Additionally, Dkk1 and Dkk2 are reported to have significantly different binding affinities for LRP6, with *K*_d_s of 0.34 and 0.7 nM, respectively^15^.

**Figure 3:**
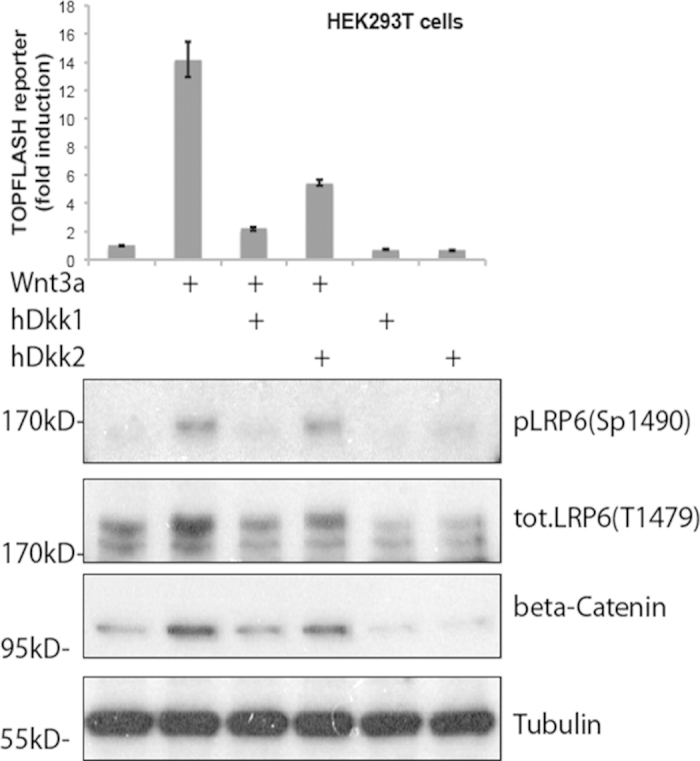
Comparison of Dkk1/2-LRP6 interaction and Wnt signaling inhibition. Wnt reporter assay (top graph) and western blots showing endogenous Wnt pathway protein components (lower panel) in HEK293T cells treated with control medium or conditioned medium containing mouse Wnt3a, human Dkk1-GFP or human Dkk2-GFP, as indicated. 12 h after transfection with the TOPFLASH reporter plasmid construct, cells were incubated with Wnt3a or control conditioned medium for 6 h. Cells were then incubated with Dkk1/2-GFP conditioned medium as indicated for another 12 h before harvest of cell lysates for the luciferase assay. Bars show the enhancement of Wnt activity over the untreated control sample (leftmost bar set to 1); error bars represent the standard deviation from three independent experiments. In the western blots, LRP6 phosphorylation (P-LRP6) and β-catenin protein levels were assessed using specific antibodies; tubulin was used as a normalization control.

To study the interactions of Dkk1 and Dkk2 with LRP6 in living cells using 2c2f lsFCS, we employed a fusion protein of human LRP6 with the red fluorescent protein mCherry (LRP6-mCherry), and fusion proteins of human Dkk1 and Dkk2 with enhanced GFP (Dkk1-GFP and Dkk2-GFP, respectively), as described in Materials and Methods. The secreted Dkk1-GFP and Dkk2-GFP fusion proteins were harvested with the culture medium of transfected cells (Supplementary Information, Fig. S6) and referred to as Dkk conditioned medium. A GFP antibody was used to confirm that the Dkk-GFP fusion proteins present in the conditioned medium were intact and not subjected to significant proteolytic cleavage of the GFP entity from Dkk (Supplementary Information, Fig. S7). The biological activity of the Dkk conditioned media was characterized using an established Wnt/β-catenin transcriptional reporter assay (TOPFLASH) as well as immunoblot detection of β-catenin levels (Fig. 3). In addition to these downstream pathway read-out assays for Wnt/β-catenin signaling activity, the activity of the LRP6 receptor was also directly analyzed. For this purpose, a phospho-specific antibody (Sp1490) was used that detects phosphorylated PPSP motifs within the intracellular domain of LRP6, which correlates positively with the activation state of the receptor^18^ (Fig. 3). As expected, addition of Wnt3a-conditioned medium activated Wnt/β-catenin transcriptional activity in HEK293T cells previously transfected with the TOPFLASH reporter (Fig. 3, upper graph), and also resulted in the upregulation of β-catenin levels as well as the activity of LRP6 (Fig. 3, lower panels). The Dkk1/2-GFP conditioned media inhibited all of these effects, demonstrating the functional activity of the GFP tagged Dkk proteins (Fig. 3, upper graph). In agreement with a previous study, Dkk1 displayed stronger inhibition of Wnt/β-catenin signaling activity than Dkk2^15^. Upon activation of the Wnt pathway, aggregation of activated LRP6 receptors into so-called signalosomes has been observed^19^. In the 2c2f lsFCS experiments presented here, there was no Wnt pathway activation present at any time during sample preparation or detection, so that LRP6 aggregation can be safely excluded.

**Figure 4:**
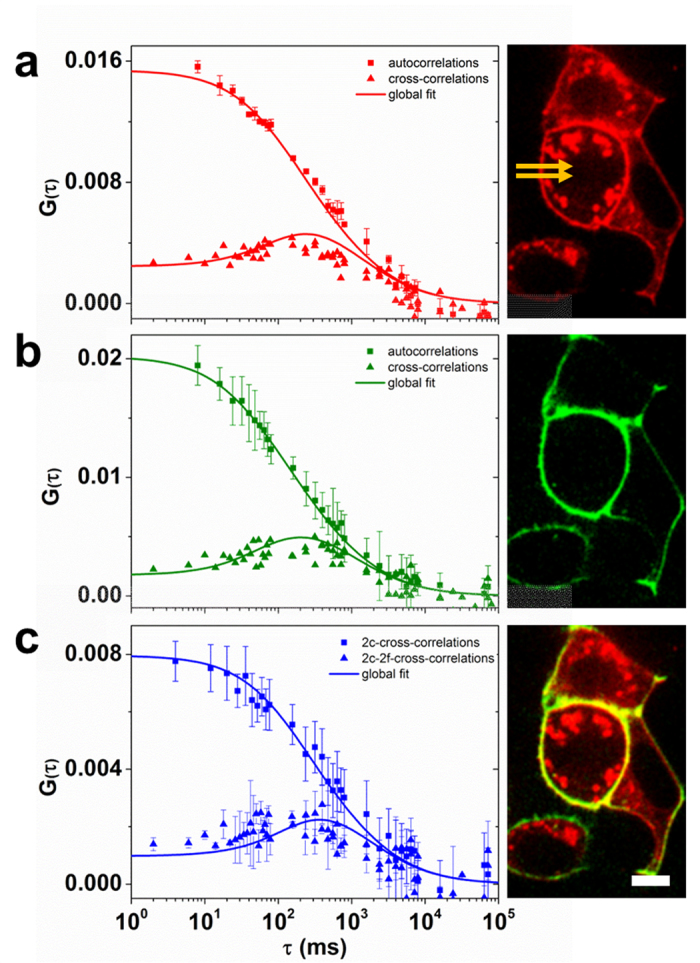
Measurement of Dkk1-GFP binding to LRP6-mCherry on HEK293T cells using 2c2f lsFCS. Left column, experimentally determined correlations functions and model fits (symbols: data, lines: fit); right column, exemplary confocal images of a cell sample. (**a**) Autocorrelation and dual-focus cross-correlation functions and image from the data collected in the red color channel; the line scan locations are marked by two yellow arrows. (**b**) Autocorrelation and dual-focus cross-correlation functions and image from the data collected in the green color channel, (**c**) dual-color cross-correlation and dual-color dual-focus cross-correlation functions and dual-color image. Error bars indicate standard deviations from multiple data sets. Scale bar, 15 μm.

For the 2c2f lsFCS experiments, HEK293T cells in 8-well chamber slides were transfected with LRP6-mCherry plasmid DNA and, 16 h later, exposed to the Dkk1/2-GFP conditioned medium (Supplementary Information, Fig. S6). After addition of Dkk-GFP, 3 min were allowed for equilibration (Supplementary Information, Fig. S8) before 2c2f lsFCS data were acquired on our home-built confocal laser scanning microscope (see Materials and Methods). Lines were scanned at positions selected from confocal images of the cells (Fig. 4, yellow arrows).

The experimental correlation curves of Dkk1-GFP binding to LRP6-mCherry are shown together with the fitted model correlation functions in Fig. 4. The autocorrelation and dual-focus cross-correlation functions in the red channel (Fig. 4a) are governed by the diffusion coefficient and the concentration of the LRP6-mCherry receptor in the membrane. The autocorrelation and two-focus cross-correlation functions in the green channel (Fig. 4b) are determined by the diffusion coefficient and the concentration of Dkk1-GFP diffusing in the membrane because of its association with the LRP6 receptor. Of note, there is an additional component to these correlation functions due to Dkk1-GFP diffusing in the extracellular medium. However, this component decays much faster and, therefore, does not contribute to the slow, millisecond time scale correlations probed by our line-scanning FCS experiment. Finally, the dual-color and dual-color dual-focus line-scanning cross-correlation data (Fig. 4c) provide information about the diffusion coefficient and the concentration of Dkk1-GFP – LRP6-mCherry ligand receptor pairs.

The global fit of all 16 2c2f lsFCS correlation curves yielded a diffusion coefficient, *D*_*R*_ = (0.18 ± 0.06) μm^2^ s^−1^, and a concentration (*i.e.*, area density in the membrane), *C*_*R*_ = (25 ± 12) μm^−2^, of the labeled receptor, and *D*_*RL*_ = (0.20 ± 0.02) μm^2^ s^−1^ and *C*_*RL*_ = (26 ± 5) μm^−2^ for the complex of labeled ligand and labeled receptor. For the unlabeled, endogenous receptor, we obtained *C*_*rL*_ = (26 ± 11) μm^−2^. Calculation of the equilibrium dissociation coefficient, *K*_*d*_, of the Dkk1-LRP6 binding reaction requires the ligand concentration to be known (Supplementary Information, Text S5). Thus, we performed an additional, static FCS experiment with the observation volume placed in the medium outside the cell, yielding a Dkk1-GFP concentration, *C*_*L*_ = (38 ± 2) nM, and a diffusion coefficient, *D*_*L*_ = (71 ± 1) μm^2^ s^−1^. We note that the RICS-like analysis of the 2c2f lsFCS data, keeping *D*_*L*_ fixed at 71 μm^2^ s^−1^, yielded *C*_*L*_ = (42 ± 2) nM, which is essentially identical to the value determined by FCS (Supplementary Information, Text S6). Consequently, an additional static FCS experiment on the live-cell sample was not necessary, assuming that *D*_*L*_ had already been determined earlier, *e.g.*, by a separate *in-vitro* FCS experiment. With *C*_*L*_ = (38 ± 2) nM from the FCS experiment, *K*_*d*_ = (37 ± 19) nM for Dkk1 binding to LRP6 (Supplementary Information, Text S5). The parameters extracted from the data in Fig. 4 are compiled in Table 1.

**Figure 5:**
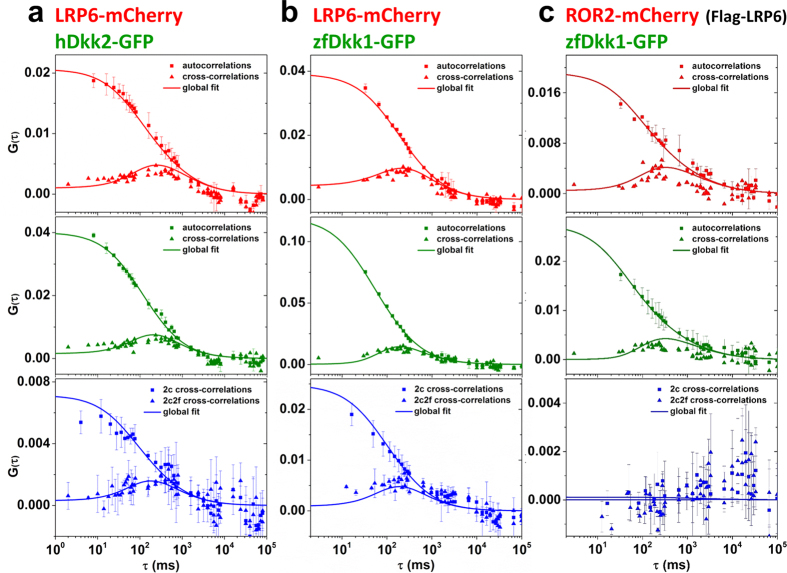
Correlation functions from 2c2f lsFCS experiments on HEK293T cells to study binding of three ligand-receptors pairs. (**a**) hDkk2-GFP binding to LRP6-mCherry, (**b**) zfDkk1-GFP to LRP6-mCherry, (**c**) zfDkk1-GFP to ROR2-mCherry. For each column, the data shown are, from top to bottom: autocorrelation and dual-focus cross-correlation functions (red channel, receptor), autocorrelation and dual-focus cross-correlation functions (green channel, ligand), and dual-color and dual-color dual-focus cross-correlation functions (plotted in blue). Error bars indicate standard deviations from multiple data sets. Symbols: experimental data, lines: fitted correlation functions.

To further test the reliability of the 2c2f lsFCS method, we studied binding of human Dkk2-GFP to LRP6. From *in-vitro* studies^15^, we expected a lower affinity for hDkk2. Indeed, representative correlation functions (data and fits) show not only high cross-correlation amplitudes, indicating significant binding, but also the expected differences in binding properties (Fig. 5 and Table 1). By contrast, zebrafish Dkk1-GFP (zfDkk1-GFP) bound to LRP6 with a similar affinity as hDkk1 (Fig. 5 and Table 1). This finding is also consistent with results from functional assays, showing that zfDkk1 suppresses Wnt pathway activation in HEK293T cells (Supplementary Information, Fig. S9).

As a negative control experiment, we co-expressed non-fluorescent LRP6 (Flag-LRP6) and an mCherry-labeled version of the receptor tyrosine kinase-like orphan receptor 2 (ROR2-mCherry), which specifically interacts with Wnt5 and functions in a pathway distinct from LRP6 mediated Wnt/β-catenin signaling^20^. We performed 2c2f lsFCS measurements on HEK293T transfected with Flag-LRP6 and ROR2-mCherry after exposing them to zfDkk1-GFP. The zfDkk1-GFP fusion protein binds to the non-fluorescent LRP in the plasma membrane, as indicated by the green staining of the membrane (not shown) and the auto- and two-focus cross-correlations in the green channel (Fig. 5, Table 1). We note that the binding of zfDkk1-GFP to endogenous LRP6 is insignificant here, since overexpressed Flag-LRP6 is far in excess (Supplementary Information, Fig. S10). In the red channel, the membrane is stained by ROR2-mCherry, and we observe the auto- and two-focus cross-correlations from receptor diffusion (Fig. 5, Table 1). Two-color cross-correlations are absent, however, so the fit yields *C*_*RL*_ = 0 ± 1 μm^−2^, *i.e*., essentially zero. It can therefore be concluded that zfDkk1 and ROR2 do not diffuse in concert and thus do not interact.

A single 2c2f lsFCS experiment with 400 s data acquisition time already yields equilibrium dissociation coefficients with satisfactory precision (Table 1). To examine the robustness of the technique and to further decrease the error margins, we have repeated the experiments several times on different samples. The equilibrium dissociation coefficient of hDkk1-GFP and LRP6-mCherry on the membrane of live cells was determined as an average (±SD) over twelve independent measurements to be (41 ± 10) nM, and the one of zfDkk1-GFP was determined from three independent measurements as (34 ± 3) nM. For hDkk2-GFP, we obtained (66 ± 11) nM from eleven independent measurements.

## Discussion

In our 2c2f lsFCS measurements, we have found different binding affinities of Dkk1 and Dkk2 toward LRP6, which correlate well with the differences observed in their ability to inhibit LRP6 phosphorylation and Wnt/β-catenin signaling (Fig. 3), with the higher-affinity Dkk1 having a stronger antagonistic effect on LRP6. Our affinity data also agree, within the error, with the previous finding that Dkk2 has a reduced affinity for LRP6 compared to Dkk1^15^. However, the dissociation coefficients that we have measured on live cells are significantly greater than in previous *in vitro* studies^14^,^15^,^16^,^17^. A strikingly similar discrepancy was previously observed when comparing FCS-based *in vivo* to *in vitro* data of FGF ligand-receptor interactions^2^. These differences may, at least in part, be attributed to the different temperatures; the *in vitro* studies of Dkk binding to LRP6 were performed at 4 °C^14^ and 20 °C,^14^,^15^,^16^ whereas the 2c2f lsFCS experiments were carried out on live cells at 37 °C. Moreover, the presence of membrane-associated proteins and an intact cellular glycocalyx and/or extracellular matrix in live cells as opposed to cellular lysates or fixed and stained cells may also give rise to different apparent affinities. This view is supported by recent FCCS studies of biomolecular affinities in live zebrafish embryos and cell cultures by Wohland and coworkers, leading them to conclude that “bimolecular interactions depend on the biological system under investigation and are best performed under physiologically relevant conditions^5^.” Finally, we note that an effect of the GFP moiety in the fusion proteins on the binding affinities cannot be excluded, neither in the *in vitro* nor in the *in vivo* experiments.

Here we have introduced 2c2f lsFCS as a robust method to directly quantify ligand binding to receptors in the plasma membranes of living cells. It exploits the advantages of the three FCS modalities utilized, and additional benefits arise from their combination. Fluorescent tags are required on both ligands and receptors. To this end, fusion constructs with fluorescent proteins of the GFP family^21^ have been employed in this study. They are very convenient tools; still, they have some adverse properties that could potentially affect the results. There always exists a fraction of non-functional (dark) proteins. For the receptor, this contribution is explicitly accounted for by the model used for the analysis. For the ligand, however, we require a maturation yield close to one for the simple reason that we cannot distinguish a receptor without a bound ligand from one that has bound a dark ligand. Fortunately, GFP-like proteins with maturation degrees of close to 100% such as enhanced GFP are available, so that this source of error can be removed. Even in their mature, fluorescence emitting form, GFP-like proteins are known to switch between dark and bright states on the microsecond time scale. This phenomenon leads to a lower effective brightness but does not affect the comparatively slow correlations monitored by 2c2f lsFCS. Another complication is the presence of permanent photodamage (bleaching) of GFP-like proteins. The line scanning approach utilized here minimizes the problem because the focal spot crosses the membrane briefly every 2 ms in our implementation, causing a very slow decay of the signal over time. In fact, we do not observe noticeable bleaching during data acquisition for typically 400 s. Bleaching and other slow fluctuations can be compensated by the perturbation correction of the intensity time traces (Supplementary Information, Text S5).

There are a number of specific advantages of 2c2f lsFCS. Because we measure dual-focus and dual-color correlations essentially simultaneously, the technique is insensitive to slow movements of the sample. It has a built-in calibration for the observation volumes of the two color and the dual-color cross-correlation channels. Moreover, it makes optimal use of the intensity data by utilizing all possible pair correlations. The experiment yields precise diffusion coefficients and concentrations of ligands outside the cell and of both unbound and ligand-bound receptors, so that ligand-receptor affinities can be determined. Importantly, we can perform these measurements on each individual cell within the field of observation in a tissue sample^22^, and even in different spots on the plasma membrane, which will enable us to explore the effects of ligand concentration gradients *in vivo* using living cells. By using appropriate scanning and analysis software, the method is easy to implement in modern laser scanning microscopes and thus may find wide application in laboratories focused on quantitative fluorescence imaging.

## Methods

### Confocal Microscope

A schematic of our homebuilt confocal (STED) microscope^23^ is shown as Supplementary Information, Fig. S11. Samples are excited by an Ar^+^ion laser (Stabilite 2017, Spectra-Physics, Mountain View, CA) and a diode-pumped solid state laser (Jive, Cobolt AB, Sweden), with emission wavelengths of 488 nm and 561 nm, respectively. The two laser beams are combined via a 540 nm long pass dichroic mirror (Q 540 LP, Chroma, Bellow Falls, VT). An acousto-optic tunable filter (AOTFnC-400.650, A-A Opto-Electronic, Orsay Cedex, France) is used for laser selection and intensity control. The excitation beam is circularly polarized by means of a quarter-wave plate (RAC 4.4.15, B-Halle, Berlin, Germany) so as to suppress photoselection of the fluorophores. After passing through a laser scanner (Yanus V, Till Photonics, Gräfelfing, Germany), the light is focused into the sample by using an oil immersion objective (HCX PL APO CS x100/1.46, Leica, Wetzlar, Germany). The fluorescence emission is collected through the same objective, separated from the excitation light by a quad band dichroic mirror (zt405/488/561/640rpc, Chroma, Bellow Falls, VT, USA) and focused into a multimode fiber (M31L02; Thorlabs, Munich, Germany) serving as a confocal pinhole. Subsequently, the fluorescence light is separated by a 555 nm longpass dichroic mirror (Q 555 LP, Chroma) and spectrally filtered by a 525/50 (center/width) nm (Brightline HC 525/50, Semrock, Rochester, NY, USA) and a 600/37 nm bandpass filter (Brightline HC 600/37, Semrock) into the two color channels. Photons are detected by avalanche photodiodes (tau-SPAD-50, PicoQuant, Berlin, Germany), and their arrival times are registered by a data acquisition card (PCI-6259, National Instruments, Munich, Germany), which also provides the signals for the laser scanner and the acousto-optic tunable filter. Data acquisition parameters, including the number of pixels and lines, pixel sizes and dwell times are controlled via homemade software written in C^++^. For static FCS measurements, the fluorescence emission is recorded with a time-correlated single photon counting (TCSPC) system (Simple-Tau 152, Becker und Hickl GmbH, Berlin, Germany) and autocorrelated using software supplied by the company. A temperature-controlled sample stage permits experiments at physiological temperature.

### FCS Data Acquisition

For a single 2c2f lsFCS measurement, data were collected for 400 s. Each scanned line consisted of 100 pixels, each one having a width of 100 nm, thus yielding a 10 μm total scan range; the separation between the two scan lines was set to 400 nm. We note that scanner fly-back and AOTF color switching may affect the pixel intensities at the beginning and end of each line scan. These data, however, are not used in the analysis. Static FCS data in the extracellular space were collected with an average acquisition time of 120 s.

### FCS Data Analysis

In the analysis software written in Matlab (MathWorks, Natick, MA), the data of the four sequential scans (two colors at two focus positions) are arranged in four two-dimensional arrays, with the pixels of each individual scan along the x-axis and the scanned lines sequentially ordered along the *y* axis. To correct for membrane movements within the confocal volume, the membrane position is determined for each line by smoothing the data with a three-pixel averaging filter and identifying the maximum intensity. Subsequently, all peaks are shifted to the same column. The average over all scan lines is computed and fitted with a Gaussian function to determine the standard deviation (width parameter) *σ*. An intensity time trace is constructed by adding up, for each line, the pixel intensities within a range of ± 2.5 *σ* from the center of the Gaussian. The auto- and cross-correlation curves of the four resulting intensity time traces are computed and globally fitted with model correlation functions by using a nonlinear least-squares fitting algorithm. From these fits, the diffusion coefficients and concentrations are obtained, so that the binding affinities can be determined. Likewise, the widths and heights of the green, red and effective observation areas are also determined in the analysis. Typical parameters for our setup are ω_g_ = (0.26 ± 0.01) μm, z_g_ = (1.22 ± 0.33) μm, ω_r_ = (0.29 ± 0.01) μm, z_r_ = (1.37 ± 0.43) μm, ω_eff_ = (0.28 ± 0.01) μm and z_eff_ = (1.30 ± 0.28) μm.

### Plasmids

Human LRP6-mCherry was generated by replacing the EGFP open reading frame (ORF) of pCS2 + hLRP6-EGFP^24^ with the ORF of mCherry, between the Xba I and SnaB I sites. The zfDkk1-GFP^25^, human Dkk1/2-GFP^26^, *Xenopus* ROR2-mCherry^27^ constructs were previously described.

### Cell Culture and Transfection

Human embryonic kidney cells (HEK293T) were maintained at 37 °C in Dulbecco’s modified eagle medium (DMEM), containing 10% fetal bovine serum (FBS), 5% CO_2_. Mouse Wnt3a conditioned medium was produced from mouse L cells stably transfected with mouse Wnt3a; control conditioned medium was from non-transfected L cells (ATCC CRL-2647 and CRL-2648, respectively). Zebrafish Dkk1-GFP and human Dkk1/2-GFP conditioned medium was produced from HEK293T cells transiently transfected with 10 μg of the corresponding plasmid per 10 cm petri dish using PromoFectin (PromoCell, Heidelberg, Germany); control conditioned medium was from pCS2-GFP transfected cells (Supplementary Information, Fig. S6).

For the FCS experiments, the following amounts of DNA per well were added to cells in 8-well slide chambers for transfection using PromoFectin: 25 ng of pCS2 + human-LRP6-mCherry, pCS2 + Flag-human-LRP6 or *Xenopus* ROR2-mCherry; 5 ng pCMV-mouse-MESD. 16 h later, different ligands were added and microscopy measurements were conducted under physiological conditions.

For TOPFLASH luciferase reporter assays and/or western blot, the following amounts of DNA per well were transfected in 96-well plates using PromoFectin: 10 ng TOPFLASH-luc and 2 ng pRp128-Rluc (Renilla control). 12 h later, 60 μl Wnt3a conditioned medium was added for Wnt signaling stimulation for 6 h. 120 μl Dkk conditioned medium was added for another 12 h inhibitory incubation before harvest.

### Luciferase Reporter Assays and Western Blot

For luciferase reporter assays, cells were harvested in 1X passive lysis buffer according to the manufacturer´s protocol (Promega, Mannheim, Germany). All samples were prepared in triplicate. TOPFLASH luciferase activity was normalized to Renilla control. All error bars shown are standard deviations from the mean of triplicates.

For western blotting, cells were harvested in 1% Triton lysis buffer (1% Triton X-100, 50 mM Tris-HCl [pH 7.0], 150 mM NaCl, 25 mM NaF, 5 mM Na_3_VO_4_, 5 mM EDTA and protease inhibitors). Western blot analysis was carried out following a standard protocol^28^, by using the following antibodies: Anti-total LRP6 (T1479^28^), PPSP phosphorylated (active) LRP6 (Sp1490, Cell Signaling Technology, Danvers, MA), total β-catenin (Sigma-Aldrich, Munich, Germany), α-tubulin (Santa Cruz Biotechnology, Heidelberg, Germany).

The secreted Dkk1-GFP fusion proteins present in the conditioned medium and used for the 2c2f lsFCS experiments were analyzed for integrity using an anti-GFP antibody (Abcam, Cambridge, UK). For the data shown in Supplementary Information, Fig. S7, 5 μl aliquots of conditioned medium were analyzed by SDS-PAGE/Western Blot.

## Author Contributions

R.M.D., Q.C., G.D. and G.U.N. designed research, R.M.D., Q.C., J.W., G.D. and G.U.N. performed research, R.M.D., Q.C., P.N.H., V.S. and M.H. contributed new reagents and analytical tools, R.M.D. and Q.C. analyzed data, R.M.D., Q.C., G.D. and G.U.N. wrote the manuscript.

## Additional Information

**How to cite this article**: Dörlich, R. M. *et al*. Dual-color dual-focus line-scanning FCS for quantitative analysis of receptor-ligand interactions in living specimens. *Sci. Rep.* **5**, 10149; doi: 10.1038/srep10149 (2015).

## Supplementary Material

Supplementary Information

## Figures and Tables

**Table 1:** Parameters obtained from a global fit to the 2c2f lsFCS data.

	**hDkk1-LRP6**	**hDkk2-LRP6**	**zfDkk1-LRP6**	**zfDkk1-ROR2**^a^
*C*_*R*_/μm^−2^	25 ± 12	38 ± 16	24 ± 8	34 ± 24
*C*_*RL*_/μm^−2^	26 ± 5	15 ± 2	15 ± 2	0 ± 1
*C*_*rL*_/μm^−2^	26 ± 11	50 ± 9	19 ± 4	0 ± 3
*D*_*R*_/μm^2^ s^−1^	0.18 ± 0.06	0.24 ± 0.06	0.18 ± 0.05	0.20 ± 0.05
*D*_*RL*_/μm^2^ s^−1^	0.20 ± 0.02	0.20 ± 0.02	0.24 ± 0.02	0.01 ± 0.13
*K*_*d*_/nM	37 ± 19	58 ± 25	34 ± 12	N/A
*C*_*L*_^b^/nM	38 ± 2	23 ± 2	21 ± 1	37 ± 2
*D*_*L*_^b^/μm^2^ s^−1^	71 ± 1	70 ± 1	70 ± 9	70 ± 9

^a^The fit returns two additional parameters for LRP6, *C*_*r2*_ = (35 ± 22) μm^–2^ and *D*_*r2L*_ = (0.22 ± 0.07) μm^2^ s^−1^.

^b^Ligand concentrations and diffusion coefficients as determined by FCS in the medium outside the cells.

## References

[b1] de Jong L. A. A., Uges D. R. A., Franke J. P. & Bischoff R. J. Receptor-ligand binding assays: technologies and applications. J. Chromatogr. B Analyt. Technol. Biomed. Life Sci. 829, 1–25 (2005). 10.1016/j.jchromb.2005.10.002 16253574

[b2] Ries J., Yu S. R., Burkhardt M., Brand M. & Schwille P. Modular scanning FCS quantifies receptor-ligand interactions in living multicellular organisms. Nat. Methods 6, 643–645 (2009). 19648917 10.1038/nmeth.1355

[b3] Tetin S. Y. Methods in Enzymology, Vol. 518 & 519. Elsevier2013). 10.1016/B978-0-12-388422-0.09990-8 23276543

[b4] Maeder C. I. *et al.* Spatial regulation of Fus3 MAP kinase activity through a reaction-diffusion mechanism in yeast pheromone signalling. Nat. Cell. Biol. 9, 1319–1326 (2007). 17952059 10.1038/ncb1652

[b5] Shi X. *et al.* Determination of Dissociation Constants in Living Zebrafish Embryos with Single Wavelength Fluorescence Cross-Correlation Spectroscopy. Biophys. J. 97, 678–686 (2009). 19619483 10.1016/j.bpj.2009.05.006 PMC2711317

[b6] Dertinger T. *et al.* Two-focus fluorescence correlation spectroscopy: a new tool for accurate and absolute diffusion measurements. ChemPhysChem. 8, 433–443 (2007). 17269116 10.1002/cphc.200600638

[b7] Schwille P., Meyer-Almes F. J. & Rigler R. Dual-color fluorescence cross-correlation spectroscopy for multicomponent diffusional analysis in solution. Biophys. J. 72, 1878–1886 (1997). 9083691 10.1016/S0006-3495(97)78833-7 PMC1184381

[b8] Bacia K., Kim S. A. & Schwille P. Fluorescence cross-correlation spectroscopy in living cells. Nat. Methods 3, 83–89 (2006). 16432516 10.1038/nmeth822

[b9] Yu S. R. *et al.* Fgf8 morphogen gradient forms by a source-sink mechanism with freely diffusing molecules. Nature 461, 533–536 (2009). 19741606 10.1038/nature08391

[b10] Ries J. & Schwille P. Studying slow membrane dynamics with continuous wave scanning fluorescence correlation spectroscopy. Biophys. J. 91, 1915–1924 (2006). 16782786 10.1529/biophysj.106.082297 PMC1544284

[b11] Digman M. A. *et al.* Measuring fast dynamics in solutions and cells with a laser scanning microscope. Biophys. J. 89, 1317–1327 (2005). 15908582 10.1529/biophysj.105.062836 PMC1366616

[b12] Clevers H. & Nusse R. Wnt/beta-catenin signaling and disease. Cell 149, 1192–1205 (2012). 22682243 10.1016/j.cell.2012.05.012

[b13] Niehrs C. Function and biological roles of the Dickkopf family of Wnt modulators. Oncogene 25, 7469–7481 (2006). 17143291 10.1038/sj.onc.1210054

[b14] Bafico A., Liu G., Yaniv A., Gazit A. & Aaronson S. A. Novel mechanism of Wnt signalling inhibition mediated by Dickkopf-1 interaction with LRP6/Arrow. Nat. Cell. Biol. 3, 683–686 (2001). 11433302 10.1038/35083081

[b15] Mao B. *et al.* LDL-receptor-related protein 6 is a receptor for Dickkopf proteins. Nature 411, 321–325 (2001). 11357136 10.1038/35077108

[b16] Semenov M. V. *et al.* Head inducer Dickkopf-1 is a ligand for Wnt coreceptor LRP6. Curr. Biol. 11, 951–961 (2001). 11448771 10.1016/s0960-9822(01)00290-1

[b17] Bourhis E. *et al.* Reconstitution of a frizzled8.Wnt3a.LRP6 signaling complex reveals multiple Wnt and Dkk1 binding sites on LRP6. J. Biol. Chem. 285, 9172–9179 (2010). 20093360 10.1074/jbc.M109.092130 PMC2838336

[b18] Tamai K. *et al.* A mechanism for Wnt coreceptor activation. Mol. Cell 13, 149–156 (2004). 14731402 10.1016/s1097-2765(03)00484-2

[b19] Bilić J. *et al.* Wnt induces LRP6 signalosomes and promotes dishevelled-dependent LRP6 phosphorylation. Science 316, 1619–1622 (2007). 17569865 10.1126/science.1137065

[b20] Oishi I. *et al.* The receptor tyrosine kinase Ror2 is involved in non-canonical Wnt5a/JNK signalling pathway. Genes Cells 8, 645–654 (2003). 12839624 10.1046/j.1365-2443.2003.00662.x

[b21] Nienhaus K. & Nienhaus G. U. Fluorescent proteins for live-cell imaging with super-resolution. Chem. Soc. Rev. 43, 1088–1106 (2014). 24056711 10.1039/c3cs60171d

[b22] Wallkamm V. *et al.* Live imaging of Xwnt5A-ROR2 complexes. PLoS ONE 9, e109428 (2014). 25313906 10.1371/journal.pone.0109428 PMC4196911

[b23] Hedde P. N. *et al.* Stimulated emission depletion-based raster image correlation spectroscopy reveals biomolecular dynamics in live cells. Nat. Commun. 4, 2093 (2013). 23803641 10.1038/ncomms3093

[b24] Mao B. *et al.* Kremen proteins are Dickkopf receptors that regulate Wnt/beta-catenin signalling. Nature 417, 664–667 (2002). 12050670 10.1038/nature756

[b25] Caneparo L. *et al.* Dickkopf-1 regulates gastrulation movements by coordinated modulation of Wnt/beta catenin and Wnt/PCP activities, through interaction with the Dally-like homolog Knypek. Genes Dev. 21, 465–480 (2007). 17322405 10.1101/gad.406007 PMC1804334

[b26] Brott B. K. & Sokol S. Y. Regulation of Wnt/LRP signaling by distinct domains of Dickkopf proteins. Mol. Cell Biol. 22, 6100–6110 (2002). 12167704 10.1128/MCB.22.17.6100-6110.2002 PMC133995

[b27] Feike A. C., Rachor K., Gentzel M. & Schambony A. Wnt5a/Ror2-induced upregulation of xPAPC requires xShcA. Biochem. Biophys. Res. Commun. 400, 500–506 (2010). 20732301 10.1016/j.bbrc.2010.08.074

[b28] Davidson G. *et al.* Casein kinase 1 gamma couples Wnt receptor activation to cytoplasmic signal transduction. Nature 438, 867–872 (2005). 16341016 10.1038/nature04170

