# Supplementary material for: Dual-color dual-focus line-scanning FCS for quantitative analysis of receptor-ligand interactions in living specimens
Source: Sci Rep. 2015 May 7;5:10149. doi: 10.1038/srep10149 (PMC4423563; doi:10.1038/srep10149)
Supplement: Supplementary Information [file srep10149-s1.pdf]

# Supplementary Information

## Dual-color dual-focus line-scanning FCS for quantitative analysis of receptor-ligand interactions in living specimens

René M. Dörlich[1,†], Qing Chen[2,3,†], Per Niklas Hedde[1], Vittoria Schuster[1], Marc Hippler[1], Janine Wesslowski[2], Gary Davidson[2,*] and G. Ulrich Nienhaus[1,2,4,*]

[1]Institute of Applied Physics, Karlsruhe Institute of Technology (KIT), 76049 Karlsruhe, Germany.

[2]Institute of Toxicology and Genetics, Karlsruhe Institute of Technology (KIT), 76021 Karlsruhe, Germany.

[3]Faculty of Bioscience, University of Heidelberg, 69120 Heidelberg, Germany.

[4]Department of Physics, University of Illinois at Urbana-Champaign, Urbana, IL 61801, USA.

**Text S1: Fluorescence correlation spectroscopy (FCS)**

In FCS experiments[1], intensity fluctuations due to fluorescent molecules diffusing through a static observation volume are continuously monitored over time (**Fig. S1**). From these intensity traces, the autocorrelation function, $G(\tau)$, is computed for different lag times $\tau$,

$$G(\tau) = \frac{\langle \delta F(t) \delta F(t+\tau) \rangle}{\langle F(t) \rangle^2}. \qquad \text{[S1]}$$

$F(t)$ is the fluorescence intensity at time $t$, and $\delta F(t) = F(t) - \langle F(t) \rangle$ is its deviation from the mean. For free diffusion of fluorescent molecules in three dimensions through a 3D Gaussian-shaped confocal observation volume, a rather simple expression results for the autocorrelation function,

$$G(\tau) = \frac{1}{\langle N \rangle} \left(1 + \frac{\tau}{\tau_d}\right)^{-1} \left(1 + \frac{\omega_0^2}{z_0^2} \frac{\tau}{\tau_d}\right)^{-1/2} \left(1 + K e^{-\tau/\tau_K}\right). \qquad \text{[S2]}$$

By fitting the experimental data with this model function, two parameters can be extracted. The amplitude at $\tau = 0$, $G(0)$, is the inverse of the average number of particles in the confocal volume, $\langle N \rangle = CV_{eff}$, with the effective observation volume, $V_{eff} = \pi^{3/2} \omega_0^2 z_0$, from which the fluorophore concentration, $C$, can be calculated. In the definition of the effective volume, the lateral ($x$, $y$) and axial ($z$) extensions, $\omega_0$ and $z_0$, are defined as the distances from the center, for which the Gaussians have decreased by a factor of $1/e^2$. The temporal decay of $G(\tau)$ is governed by the correlation time, $\tau_d = \omega_0^2/4D$, with diffusion coefficient *D*. The last term in Eq. S2 accounts for intrinsic intensity fluctuations of the fluorophore, *e.g.*, due to intersystem crossing or protonation/deprotonation. Here, $K$ describes the amplitude of these fluctuations; the characteristic time of these fluctuations is given by $\tau_K$. Usually, the parameters $\omega_0$ and $z_0$ of the confocal volume are determined by a reference measurement with a fluorophore of known diffusion coefficient *D*.

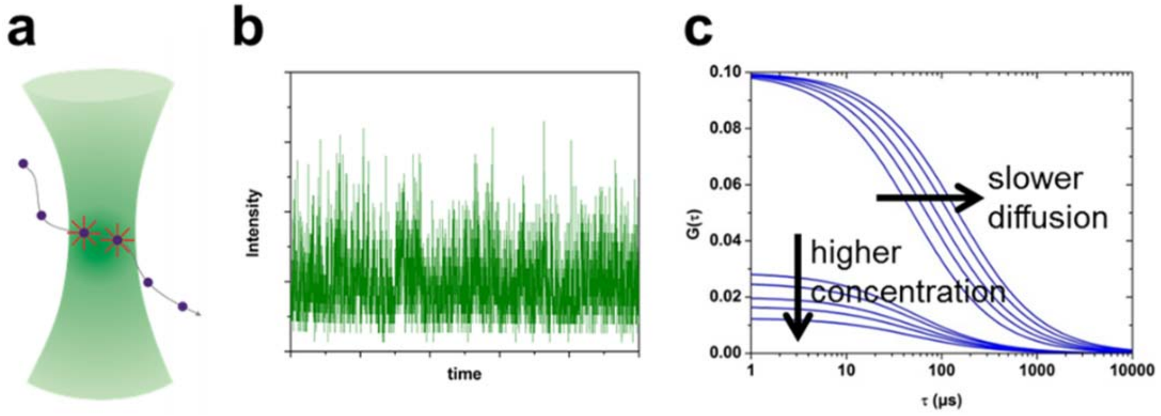


**Fig. S1: Principle of FCS.** (**a**) Fluorescent molecules give rise to intensity fluctuations when diffusing through the observation volume of a confocal microscope. (**b**) These fluctuations are monitored over time, and (**c**) an auto-correlation function is computed from these data. From the amplitude and the decay time, the concentration and the diffusion coefficient can be calculated if the size of the observation volume is known.

**Text S2: Line-scanning FCS (lsFCS)**

Conventional FCS experiments with a static confocal volume are not well suited for measuring diffusion of molecules in cell membranes because movements of the entire cell membrane within the volume will cause intensity fluctuations that can entirely obscure the fluctuations due to molecules diffusing within the membrane, which the FCS method aims to analyze. Moreover, the slow diffusion of fluorescent molecules in membranes increases the probability of photobleaching. Schwille and coworkers[2] have introduced line-scanning FCS to alleviate these problems. In this approach, the observation volume is repeatedly raster-scanned perpendicularly through a membrane (**Fig. S2**). The data can be visualized as a kymogram, in which the intensity is plotted as a function of the scanner position along the horizontal axis for each individual line scan. Lines from individual scans are plotted sequentially, each new scan below the previous one, so that the vertical dimension represents the scan number (and thus also the overall measurement time). Due to membrane fluctuations, the intersection of the confocal volume with the membrane will generally be different for each individual scan. Locating the maximum in each individual scan allows one to shift the data from all scans to a common time origin. A corrected intensity time trace, $F(t)$, results, from which the

autocorrelation curve, $G(\tau)$, can be calculated with Eq. S1. For a line scan along the (lateral) $x$-axis, the model autocorrelation function,

$$G(\tau) = \frac{1}{CA_{eff}}\left(1+\frac{4D\tau}{\omega_0^2}\right)^{-1/2}\left(1+\frac{4D\tau}{z_0^2}\right)^{-1/2}, \quad [S3]$$

applies to two-dimensional diffusion within the membrane along the *y*- and *z*-directions. Here, we again assume a Gaussian shape of the observation volume, and $\langle N \rangle = CA_{eff}$ , with concentration $C$ and effective observation area, $A_{eff} = \pi\omega_0 z_0$ . The time resolution of lsFCS is given by the duration of a single scan line, *i. e.*, the pixel dwell time times the total number of pixels.

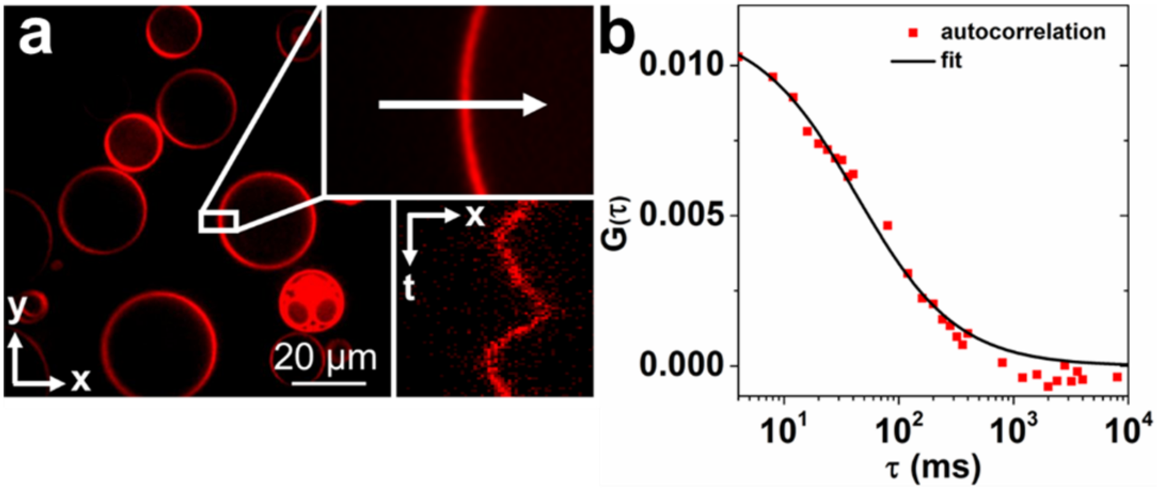


**Fig. S2: Principle of lsFCS.** (**a**) Overview and close-up image of giant unilamellar vesicles labeled with Atto590-DPPE and Atto647N-DPPE. Here, we show only the fluorescence in the Atto647N channel. In lsFCS measurements, the observation volume is scanned (white arrow in the close-up) in a direction perpendicular to the membrane (red). The scanned lines are arranged consecutively in an *x*-*t* kymogram, and the individual scans are shifted to a common time origin. (**b**) The autocorrelation function due to fluorophores diffusing within the membrane is calculated from the extracted intensity time trace and fitted with Eq. S3.

**Text S3: Dual-focus lsFCS**

In standard FCS experiments, the spatial extension of the observation volume has to be precisely known to determine concentrations and diffusion coefficients. Enderlein and coworkers[3] have introduced dual-focus FCS to circumvent the need for a separate calibration experiment. In dual-focus lsFCS, two parallel lines having a well-defined displacement, *d*, are scanned in an alternating fashion (**Fig. S3**). From the intensity time traces, the autocorrelation ($i = j$, with $i$ = 1, 2) and cross-correlation ($i \neq j$) functions are computed,

$$G_{ij}(\tau) = \frac{\langle \delta F_i(t) \delta F_j(t+\tau) \rangle}{\langle F_i(t) \rangle \langle F_j(t) \rangle}. \qquad \text{[S4]}$$

We note that the spatial displacement has to be small enough to ensure that there is an appreciable probability to collect photons from the same molecule during both scans. By scanning two lines along the *x*-direction with a displacement *d* along the *y*-direction, we can measure free diffusion of molecules in the *yz*-plane of the membrane. The model function of the two-focus pair-correlation is given by

$$G_{ij}(\tau) = G_{ji}(\tau) = \frac{1}{CA_{eff}} \left(1 + \frac{4D\tau}{\omega_0^2}\right)^{-1/2} \left(1 + \frac{4D\tau}{z_0^2}\right)^{-1/2} \exp\left(-\frac{d^2}{\omega_0^2 + 4D\tau}\right). \qquad \text{[S5]}$$

For the autocorrelation functions from both scans, $G_{ii}(\tau)$ and $G_{jj}(\tau)$, $d = 0$, so that Eq. S5 becomes identical to Eq. S3, whereas the cross-correlation function ($i \neq j$) contains the additional exponential term. By globally fitting the two autocorrelation and the cross-correlation functions, the parameter $\omega_0$ can be extracted from the data. We note that a dual-focus experiment with line displacement along *z* yields the parameter $z_0$. In practice, the ratio $\omega_0/z_0$ is a well-controlled parameter and, therefore, such a measurement is typically not required.

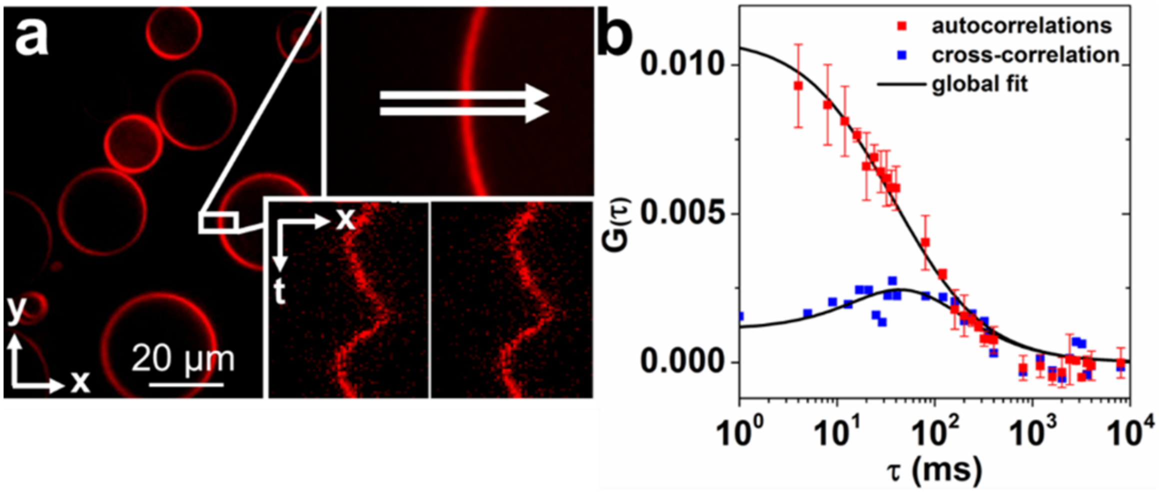


**Fig. S3: Principle of dual-focus lsFCS.** (**a**) Overview and close-up image of giant unilamellar vesicles labeled with Atto590-DPPE and Atto647N-DPPE. Here, only data from the Atto647N channel are shown. In dual-focus lsFCS measurements, the two observation volumes, with a fixed displacement, *d*, are scanned in an alternating fashion (white arrows in the close-up) perpendicularly to a vertical membrane (red). The consecutively scanned lines are arranged in two *x*-*t* images and shifted to a common time origin. (**b**) The autocorrelation (average of the data from both line scans) and cross-correlation curves of the extracted intensity traces from fluctuations in the membrane are calculated and fitted with the model correlation functions, Eqs. S3 and S5, respectively.

**Text S4: Dual-color lsFCS**

Dual-color FCS is a cross-correlation method to reveal if two differently labeled molecules bind to each other and thus diffuse as an entity[4]. Here, the fluorescence from the two molecules is monitored in two separate color channels. Assuming that cross-talk between the channels is absent (see below), a cross-correlation between the two channels is only observed if the two binding partners (*e.g.*, receptor and ligand) diffuse together (**Fig. S4**). It is important to consider that the sizes of the observation volumes associated with the two color channels are in general different. The dual-color cross-correlation function between the two color channels, $G_{r,g}(\tau)$, with labels *r* for red and *g* for green, is given by

$$G_{r,g}(\tau) = \frac{1}{\langle C\rangle A_{eff}}\left(1+\frac{4D\tau}{\frac{\omega_r^2+\omega_g^2}{2}}\right)^{-1/2}\left(1+\frac{4D\tau}{\frac{z_r^2+z_g^2}{2}}\right)^{-1/2} . \quad \text{[S6]}$$

Here, $\omega_r$ ($\omega_g$) and $z_r$ ($z_g$) are the radial and axial extensions of the red (green) observation areas, respectively. The effective detection area for dual-color cross-correlation is given by $A_{eff} = \pi\omega_{eff}z_{eff}$, with effective observation areas $\omega_{eff}^2 = (\omega_g^2 + \omega_r^2)/2$ and $z_{eff}^2 = (z_g^2 + z_r^2)/2$. Artificial, false-positive cross-correlations can arise from spectral cross-talk. This problem is avoided by using alternating excitation, so that every other line is scanned with a different color[2]. In this way, the emission from the two fluorophores can be completely separated.

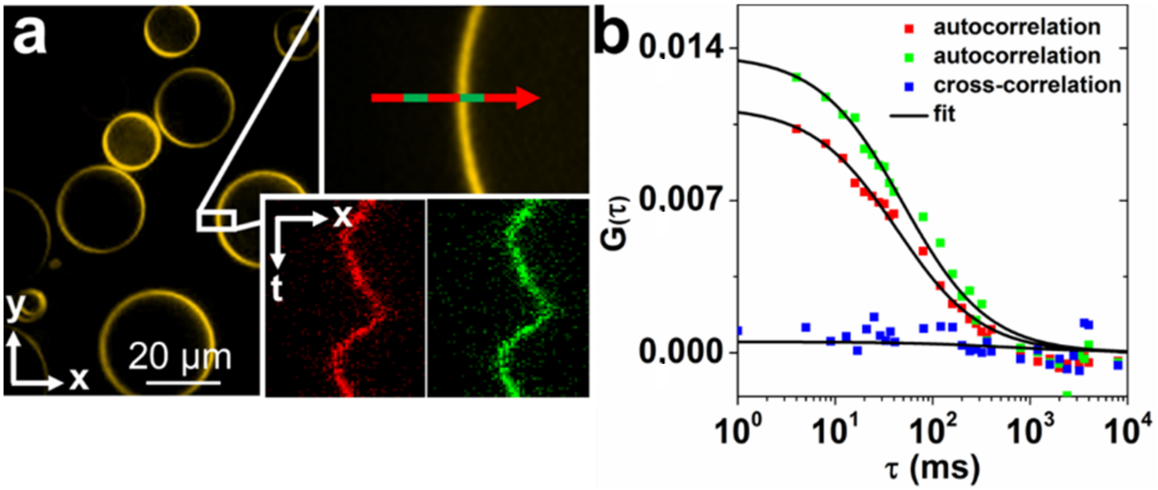


**Fig. S4: Principle of dual-color lsFCS.** (**a**) Overview and close-up image of giant unilamellar vesicles labeled with Atto590-DPPE and Atto647N-DPPE. The observation volumes are alternatingly scanned (red-green arrow in the close-up) perpendicularly through a vertical membrane (yellow) so as to avoid cross-talk. The consecutively scanned lines are arranged in two *x*-*t* images and shifted to a common time origin. (**b**) The autocorrelation (green and red squares) and cross-correlation functions (blue squares) of the extracted intensity traces from molecules diffusing in the membrane can be calculated and fitted with Eqs. S3 and S6. In this experiment, the cross-correlation amplitude is zero, indicating that the differently labeled DPPE molecules do not bind appreciably to each other.

## Text S5: Implementation of the 2c2f lsFCS method

### Principle of 2c2f lsFCS

In 2c2f lsFCS, the observation volumes are repeatedly scanned perpendicularly through a vertical membrane, using a scan sequence consisting of four consecutive scans, *i.e.*, two parallel line scans for two spectral channels (**Fig. 2a**). Spectral cross-talk is completely avoided by selecting only photons from one or the other color channel to calculate the intensity trace. The key advantage of this approach is the simultaneous assessment of the observation volume sizes of both spectral channels and the measurement of the receptor and complex concentrations with high statistical accuracy and short measurement time.

After evaluating the four intensity traces, a total of 16 autocorrelation and cross-correlation curves are calculated with Eq. S4 (**Fig. 2b**). The expressions for the autocorrelation functions and the dual-focus and dual-color cross-correlation functions are given by Eqs. S3, S5 and S6, respectively. In 2c2f lsFCS, we analyze the intensity pair correlations of red focus 1 with green focus 2 and red focus 2 with green focus 1 in addition. Assuming different observation volume sizes for the two color channels and a spatial distance *d* between the scan lines, we obtain the following model function for freely diffusing molecules in two dimensions, with one direction along the optical axis,

$$G(\tau) = \frac{1}{\langle C \rangle A_{eff}} \left(1 + \frac{4D\tau}{\omega_{eff}^2}\right)^{-1/2} \left(1 + \frac{4D\tau}{z_{eff}^2}\right)^{-1/2} \exp\left(-\frac{d^2}{4D\tau + \omega_{eff}^2}\right). \qquad [S7]$$

Note that, by setting $d = 0$, we recover the dual-color cross-correlation function (Eq. S6). By globally fitting the data (**Fig. 2c**), the receptor and complex concentrations, diffusion coefficients and sizes of the observation volumes can be determined.

### Background correction

Ligands in the extracellular space diffuse freely; these movements do not give rise to correlations on the millisecond time scale of line-scanning FCS but still contribute as an uncorrelated background to the signal in the (green) ligand channel and, thereby, affect the correlation amplitudes. To remove this background, we calculate the mean pixel intensity, $B_g$, by averaging over all pixels outside the cell. The total intensity, $F_{total}$, from the membrane is determined from the pixel intensities of the line scan by fitting a Gaussian of width σ, which is calculated in terms of the number of pixels, *p*. The

corrected intensity trace with contributions only from the membrane, $F_{\mathrm{membrane}}$, is then given by

$$F_{membrane} = F_{total} - \frac{p}{2} B_g \, . \qquad \text{[S8]}$$

A factor of 1/2 has to be included here because the free ligand is present only in the extracellular space.

**Perturbation correction**

In FCS experiments on biological specimens, many perturbations exist that may render an intensity time trace unusable for correlation analysis, including photobleaching, intensity fluctuations due to sample changes or laser instabilities, and fluorescence emitting entities that may transiently attach to the membrane. Instead of completely discarding such data, they often can be corrected, which can help to improve data statistics. For example, Ries et al.[5] used an exponential fit to account for photobleaching, whereas Lange et al.[6] recently reported a sophisticated wavelet-based approach. In our analysis, we have employed a simple yet flexible procedure that can be used on a wide variety of perturbations including photobleaching. The obvious criterion for such a correction is that it should not affect the results, *i.e.*, the procedure must not modify fluctuations on times that support the observed correlation and thus be able to recover the undisturbed intensity trace. We have found a sum-of-sines model function useful for this purpose,

$$x(t) = \sum_{i=1}^{N} a_i sin(b_i t + c_i) + d_i \, . \qquad \text{[S9]}$$

Here, $a_i$ is the amplitude, $b_i$ the frequency, $c_i$ a constant phase and $d_i$ an offset. The number of sine functions is given by $N$. By least-squares fitting the intensity time trace, $F(t)$, with this model function and subsequently dividing by the fit result, the corrected intensity time trace can be extracted,

$$F_{corr}(t) = \frac{F(t)}{x(t)} \, . \qquad \text{[S10]}$$

Depending on the length of the trace and the nature of the perturbation, the number of oscillations, $N$, has to be suitably adjusted. An excessive number of sine functions will cause the model function to suppress diffusional fluctuations and thus distort the results. We tested this approach thoroughly in experiments with GUVs to assess its reliability. In time traces without obvious artifacts, the results of the corrected and uncorrected

intensity traces were identical within the statistical error. By applying the correction to data showing clear perturbations, correlation data identical to those from unperturbed data were usually recovered. In our experiments, with typically 50,000 data points in an intensity trace, we found a sum of eight sine functions most suitable.

**Determination of equilibrium dissociation coefficients of LRP6-Dkk binding**

Here we have studied HEK293T cells expressing mCherry labeled and unlabeled LRP6 receptors in their plasma membranes; their concentrations are denoted by $C_R$ and $C_r$, respectively, in the ligand-free form. They can bind Dkk-GFP ligands, with concentration $C_L$ in the external medium, to yield receptor-ligand complexes ($C_{RL}$, $C_{rL}$). For such a bimolecular binding reaction, assuming that there is no association of the free ligand to the membrane, we can globally fit our FCS data by using the following equations for the correlation curves involving the red, green and both color channels,

$$G_r(\tau) = \frac{1}{(\langle C_R \rangle + \langle C_{RL} \rangle)^2 A_r} \times \left( \langle C_R \rangle \left(1 + \frac{4D_R\tau}{w_r^2}\right)^{-1/2} \left(1 + \frac{4D_R\tau}{z_r^2}\right)^{-1/2} \exp\left(-\frac{d^2}{4D_R\tau + w_r^2}\right) + \langle C_{RL} \rangle \left(1 + \frac{4D_{RL}\tau}{w_r^2}\right)^{-1/2} \left(1 + \frac{4D_{RL}\tau}{z_r^2}\right)^{-1/2} \exp\left(-\frac{d^2}{4D_{RL}\tau + w_r^2}\right)\right) \quad \text{[S11]}$$

$$G_g(\tau) = \frac{1}{(\langle C_{rL} \rangle + \langle C_{RL} \rangle) A_g} \times \left(1 + \frac{4D_{RL}\tau}{w_g^2}\right)^{-1/2} \left(1 + \frac{4D_{RL}\tau}{z_g^2}\right)^{-1/2} \exp\left(-\frac{d^2}{4D_{RL}\tau + w_g^2}\right) \quad \text{[S12]}$$

$$G_x(\tau) = \frac{1}{(\langle C_R \rangle + \langle C_{RL} \rangle)(\langle C_{rL} \rangle + \langle C_{RL} \rangle) A_{eff}} \times \left( \langle C_{RL} \rangle \left(1 + \frac{4D_{RL}\tau}{w_{eff}^2}\right)^{-1/2} \left(1 + \frac{4D_{RL}\tau}{z_{eff}^2}\right)^{-1/2} \exp\left(-\frac{d^2}{4D_{RL}\tau + w_{eff}^2}\right)\right) \quad \text{[S13]}$$

Here, $G_r(\tau)$ ($G_g(\tau)$) is the autocorrelation or dual-focus cross-correlation function of the red (green) channel; for the autocorrelation, $d$ = 0. $G_x(\tau)$ is the dual-color or dual-color dual-focus cross-correlation function; for the former, $d$ = 0. $D_R$ and $D_{RL}$ are the diffusion coefficients of the receptor without and with bound ligand, respectively. $A_r$ and $A_g$ are the confocal observation areas of the red and green channels, respectively, and $A_{eff}$ is the effective area in the dual-color experiment, *i.e.*, the quadratic average.

The concentrations of labeled and unlabeled receptor ($C_R$, $C_r$) as well as receptor-ligand complexes ($C_{RL}$, $C_{rL}$) are obtained from a global fit of Eqs. S11 – S13 to the 2c2f lsFCS data. In the following, we show that the equilibrium dissociation coefficient, $K_d$, can be calculated with these data plus additional information of the free ligand concentration, $C_L$. Although EGFP matures to close to 100%,[7] we may assume here for reasons of generality that there is a fraction of Dkk-GFP ligands with functional fluorophore, $C_L$, and another one that is nonfluorescent, $C_l$. Thus,

$$K_d = \frac{(C_R + C_r)(C_L + C_l)}{C_{RL} + C_{Rl} + C_{rL} + C_{rl}} \ . \quad \text{[S14]}$$

We assume that the mCherry protein fused to the receptor does not perturb its ligand binding affinity, so that

$$\frac{C_{RL} + C_{Rl}}{C_R} = \frac{C_{rL} + C_{rl}}{C_r} . \quad \text{[S15]}$$

With equation S15, we eliminate ($C_{rL}$ + $C_{rl}$) from Eq. S14,

$$K_d = \frac{(C_R + C_r)(C_L + C_l)}{C_{RL} + C_{Rl} + \frac{C_r}{C_R}(C_{RL} + C_{Rl})} \ . \quad \text{[S16]}$$

Eq. S16 can be further simplified to read

$$K_d = \frac{C_R(C_L + C_l)}{C_{RL} + C_{Rl}} \ . \quad \text{[S17]}$$

Furthermore, we assume that the GFP protein fused to the ligand does not perturb its receptor binding affinity,

$$\frac{C_l}{C_L} = \frac{C_{Rl}}{C_{RL}} . \quad \text{[S18]}$$

Upon solving Eq. S18 for $C_{Rl}$ and plugging this expression into Eq. S17, we obtain

$$K_d = \frac{C_R(C_L + C_l)}{C_{RL} + \frac{C_l}{C_L} C_{RL}} \ , \quad \text{[S19]}$$

which can be simplified to yield the standard law of mass action expression,

$$K_d = \frac{C_R C_L}{C_{RL}} \;. \tag{S20}$$

In conclusion, as long as ligand-receptor binding is not affected by the presence of fluorescent protein domains, the equilibrium dissociation coefficient, $K_d$, can be calculated on the basis of the concentrations of the fluorescent species, and does not hinge on knowing the fraction of fusion proteins that carry a functional fluorescent protein. However, we stress that we still require ligand labeling efficiencies close to one in the 2c2f lsFCS experiment. Otherwise, we have no means to distinguish between a ligand-free receptor and one with a bound, unlabeled ligand.

**Analysis of the control experiment for examining ROR2-Dkk1 binding**

The control experiment was designed to reveal lack of binding for a ligand-receptor pair that is known not to interact. To this end, we performed 2c2f lsFCS measurements on HEK293T cells expressing mCherry labeled and unlabeled ROR2 receptors and, in addition, non-fluorescent LRP6 receptors with concentrations $C_R$, $C_r$ and $C_{r2}$, respectively. Free Dkk-GFP ligands with concentration $C_L$ in the extracellular space can associate with these receptors in the membrane and form three different receptor-ligand complexes ($C_{RL}$, $C_{rL}$ and $C_{r2L}$). The global model (Eqs. S11 – S13) has to be modified slightly to analyze the 2c2f lsFCS data taken on these cells. Equation S11 is not affected because there are no changes for the red channel; just ROR2 is fluorescently labeled instead of LRP6. In Eq. S12, which describes correlations in the green channel due to ligands bound to receptors, we model the three ligand-receptor species with their respective concentrations and the diffusion coefficients of ROR2-Dkk1, $D_{RL}$, and LRP6-Dkk1, $D_{r2L}$,

$$G_g(\tau) = \frac{1}{(\langle C_{rL}\rangle + \langle C_{RL}\rangle + \langle C_{r2L}\rangle)^2 A_g} \tag{S12A}$$

$$\times\left( (\langle C_{rL}\rangle + \langle C_{RL}\rangle)\left(1+\frac{4D_{RL}\tau}{\omega_g^2}\right)^{-1/2}\left(1+\frac{4D_{RL}\tau}{z_g^2}\right)^{-1/2}\exp\left(-\frac{d^2}{4D_{RL}\tau+\omega_g^2}\right) + \langle C_{r2L}\rangle\left(1+\frac{4D_{r2L}\tau}{\omega_g^2}\right)^{-1/2}\left(1+\frac{4D_{r2L}\tau}{z_g^2}\right)^{-1/2}\exp\left(-\frac{d^2}{4D_{r2L}\tau+\omega_g^2}\right)\right)$$

The expression for the cross-correlation, Eq. S13, also needs a modification to account for the presence of three ligand-receptor species with a green-labeled ligand,

$$G_x(\tau) = \frac{1}{(\langle C_R\rangle + \langle C_{RL}\rangle)(\langle C_{rL}\rangle + \langle C_{RL}\rangle + \langle C_{r2L}\rangle)A_{eff}} \quad \text{[S13A]}$$

$$\times\left(\langle C_{RL}\rangle\left(1+\frac{4D_{RL}\tau}{\omega_{eff}^2}\right)^{-1/2}\left(1+\frac{4D_{RL}\tau}{z_{eff}^2}\right)^{-1/2} exp\left(-\frac{d^2}{4D_{RL}\tau+\omega_{eff}^2}\right)\right)$$

Thus, the global fit returns two more parameters than the one using Eqs. S11 – 13, characterizing the second receptor, $C_{r2}$ and $D_{r2L}$.

**Text S6: Free ligand diffusion analysis from a RICS-like analysis of lsFCS data**

In FCS studies of ligand binding to cell surface receptors, the concentration and the diffusivity of the free ligand are usually measured with a conventional FCS experiment in the volume outside the cell. Here we show that we can obtain these quantities also from our lsFCS data directly; therefore, an extra FCS experiment is not required.

In raster image correlation spectroscopy (RICS[8]), a method conceptually closely related to FCS, raster-scanned images are acquired by moving the observation volume across the sample with a well-defined timing protocol defined by the pixel and line dwell times. Because the spatial relation between any two pixels can be recast into a time interval, dynamic information can be obtained from a two-dimensional spatial correlation function, which is calculated between pixels along the two scan directions,

$$G(\xi,\psi) = \frac{\langle F(x,y)F(x+\xi,y+\psi)\rangle_{x,y}}{\langle F(x,y)\rangle_{x,y}^2}. \quad \text{[S21]}$$

Here, $F(x,y)$ is the pixel intensity at position *x*, *y*; $F(x+\xi,y+\psi)$ is the fluorescence intensity at a position shifted $\xi$ pixels along the x and $\psi$ lines along the y axis, similar to the time shift $\tau$ in FCS experiments. This analysis can be applied to kymograms measured in line-scanning FCS experiments (**Fig. S5**). The computed correlation function can be fitted with

$$G(\xi,\psi) = \frac{1}{N} G_D(\xi,\psi)\, G_s(\xi,\psi)\, G_K(\xi,\psi) \;. \quad \text{[S22]}$$

Here, *N* is the average number of fluorophores within the observation volume. The term $G_D(\xi,\psi)$ accounts for free, three-dimensional diffusion,

$$G_D(\xi,\psi) = \left(1+\frac{4D(\tau_p\xi+\tau_l\psi)}{\omega_0^2}\right)^{-1}\left(1+\frac{4D(\tau_p\xi+\tau_l\psi)}{z_0^2}\right)^{-1/2} . \quad \text{[S23]}$$

The pixel and line dwell times are denoted by $\tau_\text{p}$ and $\tau_\text{l}$, respectively. In line-scanning FCS experiments, the displacement along the *y* axis, $\psi$, denotes only a temporal but not a spatial shift. Therefore, the scanning part, $G_S(\xi,\psi)$, of the correlation function is modified from the one given in Ref. 8 and given by

$$G_S(\xi,\psi) = \exp\left(-\frac{\left(\frac{2\xi\delta_p}{\omega_0}\right)^2}{2\left(1+\frac{4D\left(\tau_p\xi+\tau_l\psi\right)}{\omega_0^2}\right)}\right) , \quad \text{[S24]}$$

with the pixel size $\delta_\text{p}$ = 100 nm in our experiments. We have included a kinetic term, $G_K(\xi,\psi)$, as in Eq. S2. In our experiments, it represents emission fluctuations of the fluorescent proteins (flickering) with relaxation times, $\tau_\text{K}$, of 20 – 60 µs,

$$G_K(\xi,\psi) = 1 + K\exp\left(-\frac{\tau_p\xi+\tau_l\psi}{\tau_K}\right), \quad \text{[S25]}$$

*K* denotes the amplitude of the flickering. With a pixel dwell time of 20 µs in our line scanning experiments, we cannot accurately measure such fast processes. Here, we have included the kinetic term so as to obtain an accurate, quantitative comparison with the data at higher time resolution measured by static FCS.

In our measurements of receptor-ligand affinities, we have determined the free ligand concentration by static FCS because of the better statistical accuracy. The RICS analysis is less precise because of the poorer time resolution due to the pixel dwell time of 20 µs. With the diffusion coefficient and flickering amplitude determined in prior *in vitro* experiments on dilute solutions of the ligand, these two parameters can be fixed in the RICS-like analysis. With this approach, we have obtained free ligand concentrations that were identical within the statistical error to those obtained by FCS. With further optimization of the data acquisition parameters, pixel size and dwell time, the RICS-like analysis should become widely applicable also for *in vivo* measurements.

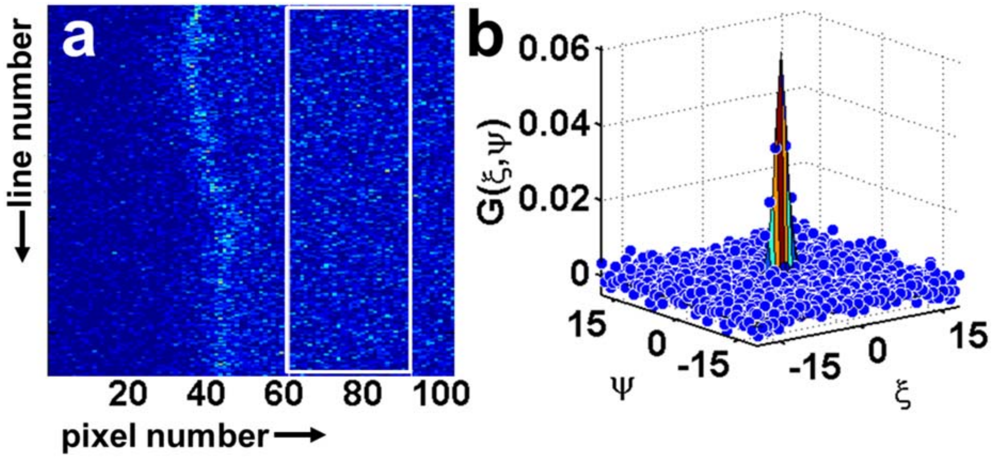


**Fig. S5: RICS-like analysis of lsFCS data.** (**a**) The fluorescence from freely diffusing Dkk1-GFP ligands outside the cell membrane is clearly visible on the right hand side of the membrane in the kymogram generated from line scanning data. (**b**) The pixels inside the area marked by the white box in panel (a) were used to calculate the experimental correlation curve (blue circles). Ligand diffusion is much faster than the line dwell time, so there are no correlations along the $\psi$ axis. The free ligand concentration can be obtained from fitting the model function $G(\xi, \psi)$ to the correlation data.

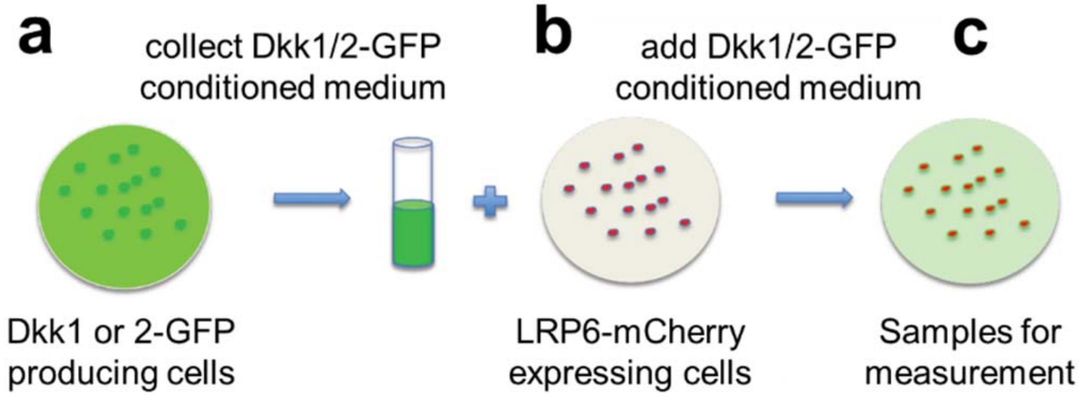


**Fig. S6: Cell sample preparation**

Samples for 2c2f lsFCS measurements on living cells were prepared as follows: **(a)** 10 µg of Dkk1/2-GFP expression plasmid was transfected into HEK293T cells in 10 cm petri dishes using PromoFectin according to the manufacturer's instruction. 48 h post transfection, the conditioned medium supernatant containing secreted Dkk1/2 molecules was harvested, centrifuged at 500g to remove any transferred cells and the clarified conditioned medium stored at 4°C for future use. **(b)** 25 ng of LRP6-mCherry (or ROR2-mCherry together with Flag-LRP6, 25 ng each) was transfected into HEK293T cells in each chamber of an 8-well slide. After 16 h, mCherry tagged receptors were expressed on the cell membrane. (**c**) GFP tagged Dkks were added to the culture medium to obtain a sample for 2c2f lsFCS measurements.

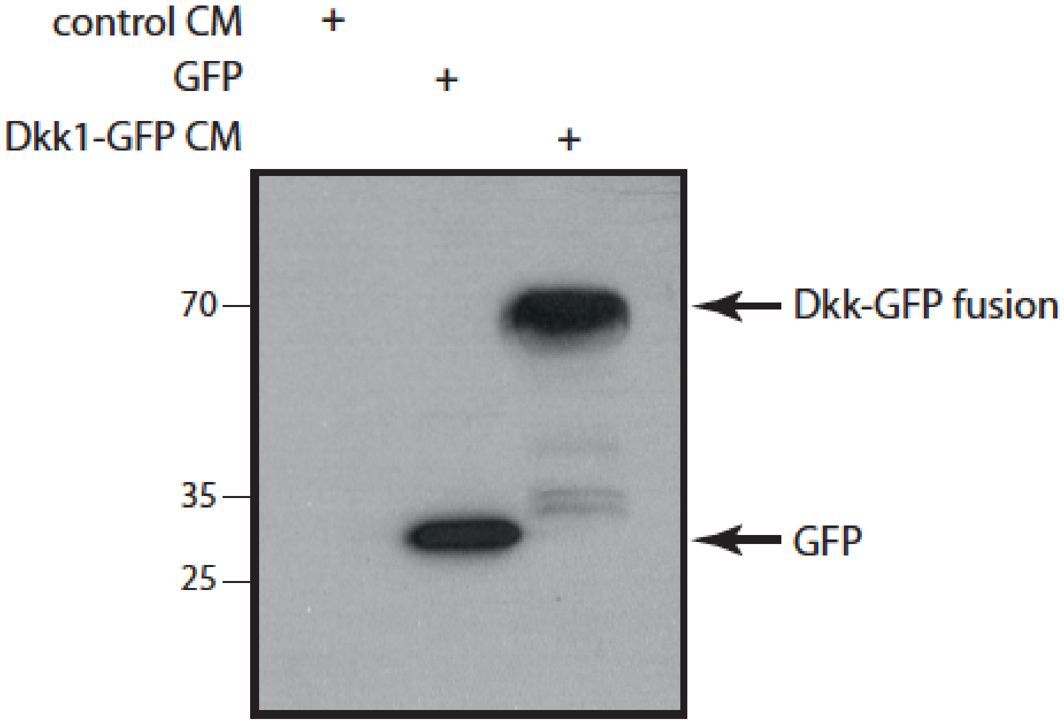


**Fig. S7: Quality control of Dkk-GFP fusion protein in conditioned medium**

Anti-GFP Western Blot of conditioned medium (CM) harvested from cells transfected with plasmid DNA constructs encoding the indicated proteins. For the GFP sample, a small aliquot of the corresponding cell lysate was loaded together with the conditioned medium in order to allow its visualization. This was done because GFP itself is a cytoplasmic protein and is not secreted from cells into the medium. For the Dkk1-GFP sample, the vast majority of GFP in the conditioned medium is associated with a 70 kDa band, which is the expected size for the Dkk1-GFP fusion protein. The GFP antibody detects only trace amounts of lower molecular weight protein fragments. Molecular weights (in kDa) are shown on the left side of the blot.

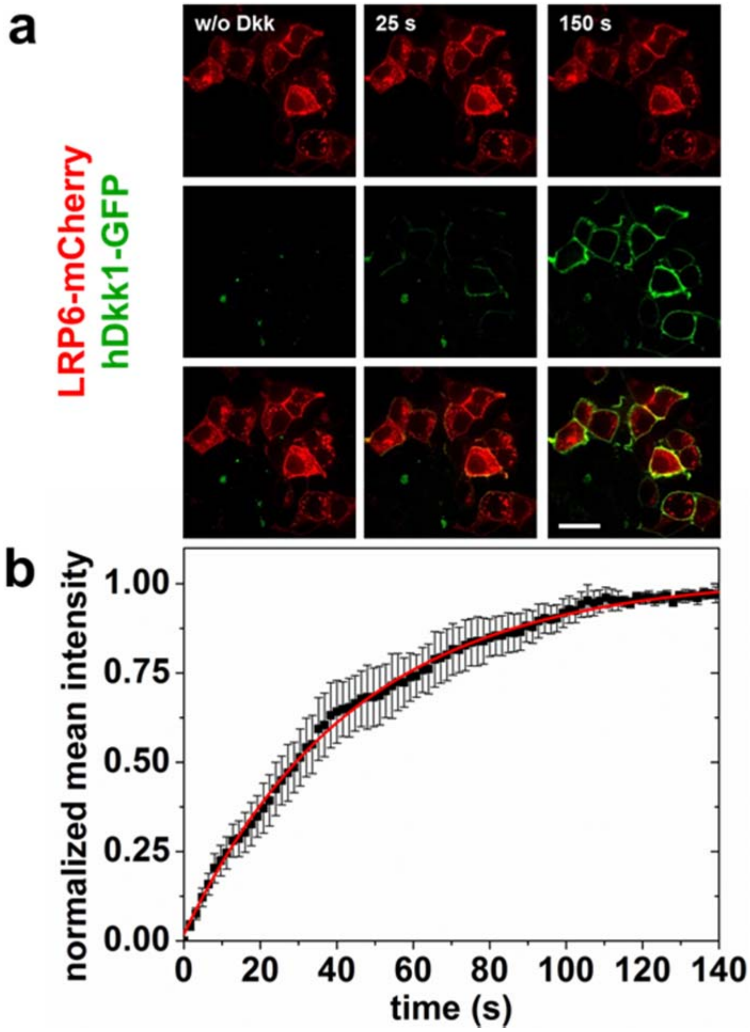


**Fig. S8: Dkk1-GFP – LRP6-mCherry equilibration time**

(**a**) Fluorescence images of LRP6-mCherry expressing HEK293T cells before and 25/150 s after addition of 30 nM of the Dkk1-GFP ligand, taken on an Andor Revolution XD spinning disk confocal microscope (BFi OPTiLAS, Munich, Germany). Top row: LRP6-mCherry fluorescence (in red), middle row: Dkk1-GFP fluorescence (in green) becomes noticeable on the cell membranes 25 s after ligand addition, bottom row: Merged images of the two color channels; scale bar: 50 µm. (**b**) Kinetic analysis of ligand binding: The normalized intensity of the Dkk1-GFP fluorescence (average over four cells in contact with the solution and not touching another cell) is plotted as a function of time. An exponential fit (red line) yields a characteristic time of (45 ± 1) s.

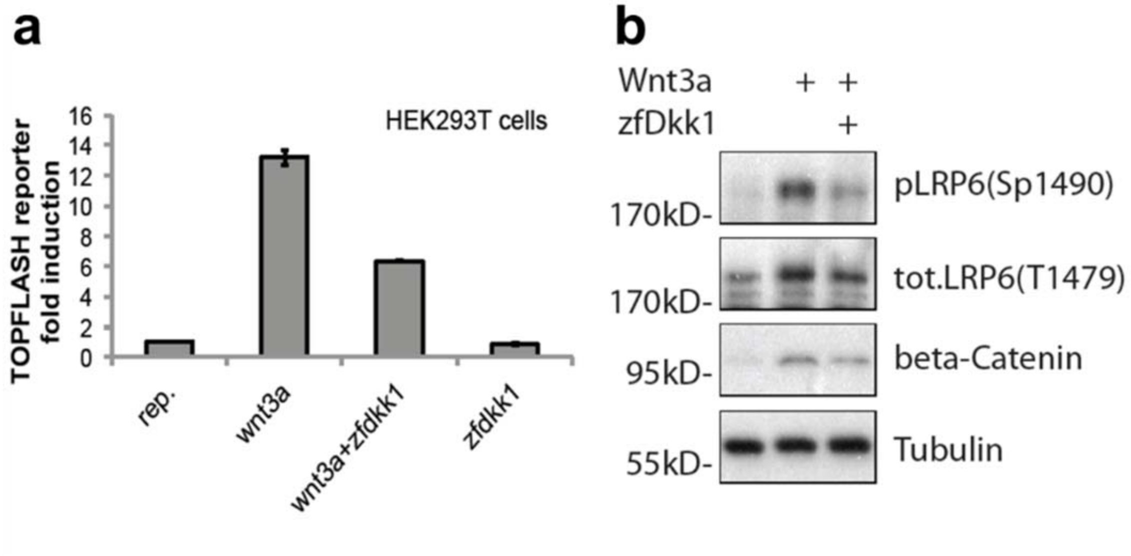


**Fig. S9: Functional characterization of zfDkk1 in Wnt signaling inhibition.**

(**a**) Reporter gene assay in HEK293T cells treated with control, mouse Wnt3a, zebrafish Dkk1-GFP (zfDkk1-GFP), or combined. Cells were first transfected with the TOPFLASH Wnt/β-catenin reporter. 12 h post transfection, Wnt3a or control conditioned medium was added for 6 h to activate the Wnt pathway. The Dkk1-GFP conditioned medium was then added as indicated for an additional 12 h before harvesting for luciferase activity measurement. Error bars represent the standard deviation of three experiments. (**b**) As an additional method to determine Wnt/β-catenin signaling activity, Western Blot analysis of the indicated proteins was performed after SDS-PAGE separation of cellular lysates, harvested from the same cells described in (**a**). Phosphorylated LRP 6 (pLRP6) was detected by an antibody recognizing residue Ser1490, when phosphorylated (Sp1490), and represents an activated form of the receptor. β-catenin accumulation itself was revealed by using a specific antibody.

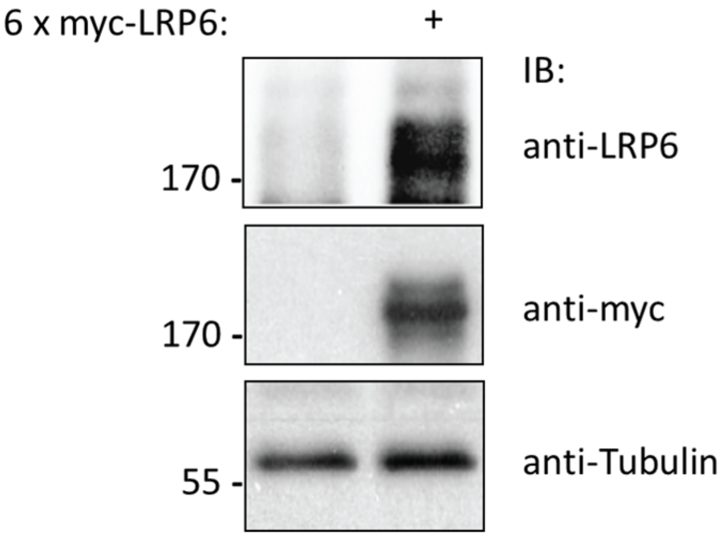


**Fig. S10: Relative levels of endogenous and overexpressed LRP6.**

Western Blots, using the indicated antibodies, show the relative levels of LRP6 proteins in lysates from untransfected HEK 293T cells or corresponding cells transfected with 6 x myc-tagged LRP6. Note that endogenous levels of LRP6 (upper panel) are almost undetectable in untransfected cells, suggesting there is only a minor contribution of endogenous LRP6 in the FCS analysis. The amount of LRP6 transfected (10 ng/96-well with a surface area of 0.32 $cm^2$) was similar to the amounts used for the FCS experiments (25 ng/well of 8-well-chambered slide with a surface area of 0.7 $cm^2$). The expression levels and functional activities of 6 x myc-LRP6, Flag-LRP6 and mCherry-LRP6 fusion proteins are similar for the same amounts transfected.

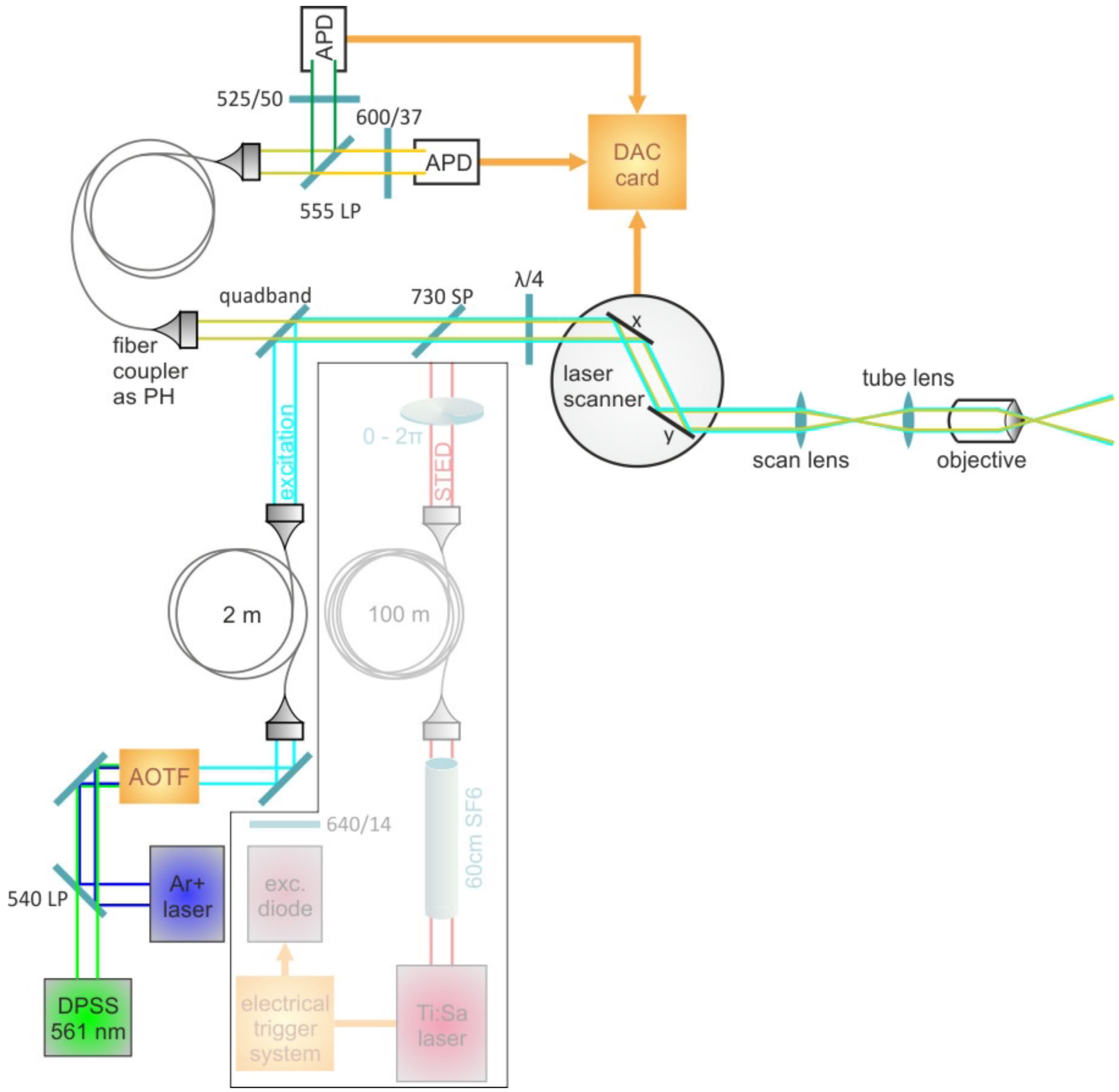


**Fig. S11: Schematic of the confocal laser scanning microscope for 2c2f lsFCS.**
Our home-built confocal laser scanning microscope setup is also capable of STED super-resolution microscopy[9]; this feature (shaded part) is not used here. A detailed description of the components is given in Materials and Methods.

## References


1. Tetin, S.Y. Methods in Enzymology, Vol. 518 & 519. (Elsevier, 2013).
2. Ries, J. & Schwille, P. Studying slow membrane dynamics with continuous wave scanning fluorescence correlation spectroscopy. *Biophys. J.* **91**, 1915-1924 (2006).
3. Dertinger, T. et al. Two-focus fluorescence correlation spectroscopy: a new tool for accurate and absolute diffusion measurements. *ChemPhysChem* **8**, 433-443 (2007).
4. Schwille, P., Meyer-Almes, F.J. & Rigler, R. Dual-color fluorescence cross-correlation spectroscopy for multicomponent diffusional analysis in solution. *Biophys. J.* **72**, 1878-1886 (1997).
5. Ries, J., Chiantia, S. & Schwille, P. Accurate determination of membrane dynamics with line-scan FCS. *Biophys. J.* **96**, 1999-2008 (2009).
6. Lange, J.J. et al. Correction of bleaching artifacts in high content fluorescence correlation spectroscopy (HCS-FCS) data. *Proc. SPIE* **8590**, 859006 (2013).
7. Sniegowski, J.A., Phail, M.E. & Wachter, R.M. Maturation efficiency, trypsin sensitivity, and optical properties of Arg96, Glu222, and Gly67 variants of green fluorescent protein. *Biochem. Biophys. Res. Commun.* **332**, 657-663 (2005).
8. Digman, M.A. et al. Measuring fast dynamics in solutions and cells with a laser scanning microscope. *Biophys. J.* **89**, 1317-1327 (2005).
9. Hedde, P.N. et al. Stimulated emission depletion-based raster image correlation spectroscopy reveals biomolecular dynamics in live cells. *Nat. Commun.* **4**, 2093 (2013).
